# Misoprostol treatment prevents hypoxia-induced cardiac dysfunction through a 14-3-3 and PKA regulatory motif on Bnip3

**DOI:** 10.1038/s41419-021-04402-3

**Published:** 2021-11-26

**Authors:** Matthew D. Martens, Nivedita Seshadri, Lucas Nguyen, Donald Chapman, Elizabeth S. Henson, Bo Xiang, Landon Falk, Arielys Mendoza, Sunil Rattan, Jared T. Field, Philip Kawalec, Spencer B. Gibson, Richard Keijzer, Ayesha Saleem, Grant M. Hatch, Christine A. Doucette, Jason M. Karch, Vernon W. Dolinsky, Ian M. Dixon, Adrian R. West, Christof Rampitsch, Joseph W. Gordon

**Affiliations:** 1 Department of Human Anatomy and Cell Science, University of Manitoba, Winnipeg, MB Canada; 2 Diabetes Research Envisioned and Accomplished in Manitoba (DREAM) Theme, Children’s Hospital Research Institute of Manitoba, Winnipeg, MB Canada; 3 Department of Physiology and Pathophysiology, University of Manitoba, Winnipeg, MB Canada; 4 Department of Biochemistry and Medical Genetics, University of Manitoba, Winnipeg, MB Canada; 5 The Research Institute in Oncology and Hematology, CancerCare Manitoba, Winnipeg, MB Canada; 6 Department of Pharmacology and Therapeutics, University of Manitoba, Winnipeg, MB Canada; 7 Biology of Breathing (BoB) Theme, Children’s Hospital Research Institute of Manitoba, Winnipeg, MB Canada; 8 Department of Molecular Physiology and Biophysics, Cardiovascular Research Institute, Baylor College of Medicine, Houston, TX USA; 9 Institute for Cardiovascular Sciences, St. Boniface Research Centre, Winnipeg, MB Canada; 10 Department of Surgery, University of Manitoba, Winnipeg, MB Canada; 11 Faculty of Kinesiology and Recreation Management, University of Manitoba, Winnipeg, MB Canada; 12 Morden Research & Development Centre, Agriculture and Agri-Food Canada, Morden, MB Canada; 13 College of Nursing, University of Manitoba, Winnipeg, MB Canada

**Keywords:** Cell death, Cardiovascular biology

## Abstract

Systemic hypoxia is a common element in most perinatal emergencies and is a known driver of Bnip3 expression in the neonatal heart. Bnip3 plays a prominent role in the evolution of necrotic cell death, disrupting ER calcium homeostasis and initiating mitochondrial permeability transition (MPT). Emerging evidence suggests a cardioprotective role for the prostaglandin E1 analog misoprostol during periods of hypoxia, but the mechanisms for this protection are not completely understood. Using a combination of mouse and cell models, we tested if misoprostol is cardioprotective during neonatal hypoxic injury by altering Bnip3 function. Here we report that hypoxia elicits mitochondrial-fragmentation, MPT, reduced ejection fraction, and evidence of necroinflammation, which were abrogated with misoprostol treatment or Bnip3 knockout. Through molecular studies we show that misoprostol leads to PKA-dependent Bnip3 phosphorylation at threonine-181, and subsequent redistribution of Bnip3 from mitochondrial Opa1 and the ER through an interaction with 14-3-3 proteins. Taken together, our results demonstrate a role for Bnip3 phosphorylation in the regulation of cardiomyocyte contractile/metabolic dysfunction, and necroinflammation. Furthermore, we identify a potential pharmacological mechanism to prevent neonatal hypoxic injury.

## Introduction

Systemic hypoxia is a major complication associated with nearly all perinatal emergencies, including placental abnormalities, preterm birth, impaired lung development, and cyanotic congenital heart disease, which are also leading causes of neonatal mortality [1]. Moreover, hypoxic injury has been shown to alter neonatal cardiac metabolism, resulting in diminished contractile performance and compromised tissue perfusion, further compounding neuro-cognitive and end-organ complications [2].

Bnip3 is a hypoxia-inducible pro-apoptotic member of the Bcl-2 family [3–7]. Bnip3 relies on a C-terminal transmembrane (TM) domain for its pro-death functions [8]. This domain inserts through the outer mitochondrial membrane to interact with optic atrophy-1 (Opa1) to promote mitochondrial fission [8–10], and can also drive mitochondrial bioenergetic collapse by affecting complexes of electron transport chain, ultimately disrupting ATP production [11]. Additionally, Bnip3 can localize to the endoplasmic reticulum (ER), interrupting Bcl-2-induced gating of the inositol trisphosphate receptor (IP_3_R), resulting in ER calcium depletion and mitochondrial matrix calcium accumulation [8, 12–15]. Elevated matrix calcium is an important trigger for mitochondrial permeability transition (MPT), a phenomena that is required for the induction of necrotic cell death [16–23]. Taken together, these observations have made Bnip3 an attractive therapeutic target in the heart, but this has been met with limited success.

Prostaglandin signaling has pleiotropic effects on the cardiovascular system [24]. Although generally regarded as pro-inflammatory, prostaglandin E1 (PGE1) has been associated with the resolution of inflammation, T cell inhibition, and wound healing [25–28]. Previous work has demonstrated that prostaglandins, acting through the EP4 receptor, activates protein kinase A (PKA) and improves cardiac function in mice following infarction [29]. Recent work from our group demonstrated that misoprostol, a PGE1 analog, is cytoprotective in cardiomyocytes exposed to hypoxia [4]. Moreover, loss of Bnip3 activity is protective in the heart following ischemia-refusion [30, 31], and work in transformed cell models identified that phosphorylation of Bnip3 within its TM domain can inhibit apoptosis [9]. However, the upstream signaling pathways that regulate post-translational modification of Bnip3 have not been previously described. In addition, it is not currently known if Bnip3 phosphorylation can be pharmacologically targeted to modulate cardiomyocyte MPT and in vivo heart function during hypoxia. Thus, we examined if misoprostol treatment is sufficient to alter Bnip3 function to prevent mitochondrial and contractile dysfunction in the neonatal heart.

In this report we provide evidence that misoprostol inhibits hypoxia-induced neonatal contractile dysfunction resulting from cardiomyocyte respiratory collapse and a necroinflammatory phenotype. We show that this is a result of inhibiting Bnip3-induced calcium transfer from the ER to the mitochondria, which prevents MPT, ATP depletion, and necrosis. Mechanistically, we demonstrate that this is regulated through EP4-mediated PKA signaling, resulting in direct phosphorylation of Bnip3 at threonine-181. We further demonstrate that the interaction between 14-3-3β and Bnip3 is increased by misoprostol treatment, facilitating Bnip3 trafficking from the ER and mitochondria. Given the diverse roles of Bnip3 in hypoxic pathologies and cancer, this mechanism may have broader implications to human disease.

## Materials and methods

### In vivo neonatal hypoxia model and adult coronary ligation model

All procedures in this study were approved by the Animal Care Committee of the University of Manitoba, which adheres to the principles for biomedical research involving animals developed by the Canadian Council on Animal Care (CCAC). N values were chosen based on predicted statistical power of 80%. No formal randomization technique was used and blinded animal studies was not possible with the use of hypoxia chambers. Litters of wild-type and/or Bnip3-null (embryonic deletion described previously [31]) B6;129 mouse pups and their dams were placed in a hypoxia chamber with 10% O_2_ (±1%) on postnatal day (PND) 3 for a period of 7 consecutive days. Control litters were left in normoxic conditions at 21% O_2_. Animals received 10 μg/kg misoprostol or saline control, administered through subcutaneous injection daily from PND3–10. At PND10 animals were euthanized and perfused with saline for tissue collection. Both male and female mice were used and results were pooled. This hypoxia protocol has been previously shown to induce cognitive impairment consistent with hypoxic-ischemic encephalopathy [32, 33]. In the in vivo rodent model of myocardial infarction, the left coronary artery of male Sprague Dawley rats was ligated approximately 2 mm from its origin, while sham operated rats serve as control [34, 35]. Following recovery for 4 or 8 weeks, animals are anesthetized, the heart excised, and the left anterior descending territory dissected for scar tissue and viable border-zone myocardium.

### In vivo assessment of cardiac function

Transthoracic echocardiography was performed on mildly anesthetized mice (sedated with 3% isoflurane in oxygen at 1 L/min and maintained at 1–1.5% isoflurane in oxygen at 1 L/min) at PND10 using a Vevo 2100 High-Resolution Imaging System equipped with a 30-MHz transducer (RMV-716; VisualSonics, Toronto) as described previously [36].

### Cell culture and transfections

Rat primary ventricular neonatal cardiomyocytes (PVNC) were isolated from 1–2-day old pups using the Pierce Primary Cardiomyocyte Isolation Kit (#88281), which includes a cardiomyocyte growth supplement to reduce fibroblast contamination. H9c2 cells (ATCC CRL-1446) were maintained in Dulbecco’s modified Eagle’s medium (DMEM; Hyclone), containing penicillin, streptomycin, and 10% fetal bovine serum (Hyclone). Media was supplemented with MEM Non-Essential Amino Acids Solution (Gibco) for MEFs. Cells were incubated at 37 °C and 5% CO2. Human induced pluripotent stem cell-derived cardiomyocytes (H-iPSC-CMs) were obtained from Cellular Dynamics (iCell Cardiomyocytes #01434). iCell Cardiomyocytes were cultured in maintenance medium as per the manufacturer’s protocol and differentiated for 72 h. Cell lines were transfected using JetPrime Polyplus reagent, as per the manufacturer’s protocol. For misoprostol treatments, 10 mM misoprostol (Sigma) in phosphate buffered saline (PBS; Hyclone) was diluted to 10 μM directly in the media and applied to cells for 24 h. To achieve hypoxia, cells were held in a Biospherix incubator sub-chamber with 1% O_2_ (±1%), 5% CO_2_, balanced with pure N_2_ (regulated by a Biospherix ProOx C21 sub-chamber controller) at 37 °C for 24 h. BvO2, L161-982, L798-106, and H89 dihydrochloride (H89) were purchased from Sigma.

### Plasmids

Mito-Emerald (mEmerald-Mito-7) was a gift from Michael Davidson (Addgene #54160) [37]. The endoplasmic reticulum (CMV-ER-LAR-GECO1), and mitochondrial (CMV-mito-CAR-GECO1) targeted calcium biosensors were gifts from Robert Campbell (Addgene #61244, and #46022) [38]. CMV-dsRed was a gift from John C. McDermott. The FRET-based ATP biosensor (ATeam1.03-nD/nA/pcDNA3) was a gift from Takeharu Nagai (Addgene plasmid #51958) [39]. The dimerization-dependent PKA biosensor (pPHT-PKA) was a gift from Anne Marie Quinn (Addgene #60936) [40]. pcDNA3-HA-14-3-3 beta (14-3-3β) was a gift from Michael Yaffe (Addgene #13270). pclbw-opa1(isoform 1)-myc (myc-Opa1) was a gift from David Chan (Addgene plasmid # 62845) [41]. The generation of mouse myc-Bnip3 (Addgene #100796) was described previously [42]. The phospho-neutral mouse myc-Bnip3-T181A was generated by PCR using the New England Biolabs Q5 Site-Directed Mutagenesis Kit and primers Forward: 5ʹ-CTAGTCTAGA ATGTCGCAGAGCGGGGAGGAGAAC-3ʹ and Reverse: 5ʹ-GATCGGATCCTCAGAAGGTGCTAGTGGAAGTtgcCAG-3ʹ.

### Fluorescent staining, live cell imaging, and immunofluorescence

MitoView Green, TMRM, Calcein-AM, ethidium homodimer-1, and Hoechst 33342 were purchased from Biotium. MitoSox was purchased from Life Technologies. MPTP imaging was described previously [19]. Dye based calcium imaging was done with Rhod-2AM (Invitrogen, R1245MP) as per manufacturer’s protocol (including the production of dihyrdorhod-2 AM). Immunofluorescence with HMBG1 (CST # 3935), Bnip3 [CST # 3769 and Ab-196706 (Alexa Fluor 647)], 14-3-3β [sc-25276 (Alexa Fluor 488)], and Opa1 [sc-393296 (Alexa Fluor 488)] antibodies were performed in conjunction with fluorescent secondary antibodies conjugated to Alexa Fluor 466 or 647 (Jackson), when primary antibodies were not conjugated to a fluorophore. All epifluorescent imaging experiments were done on a Zeiss Axiovert 200 inverted microscope fitted with a Calibri 7 LED Light Source (Zeiss) and Axiocam 702 mono camera (Zeiss) in combination with Zen 2.3 Pro imaging software. Confocal imaging was done on a Zeiss LSM700 Spectral Confocal Microscope in combination with Zen Black, which was also used for colocalization analysis, while FRET imaging was done using a Cytation 5 Cell Imaging Multi-Mode Reader. Quantification, scale bars, and processing including background subtraction, was done on Fiji (ImageJ) software. Quantification of mitochondrial morphology was performed as previously described [43].

### In vitro assessment of cellular viability

MTT assays were performed according to a protocol described previously [44]. Briefly, H9c2 cells were incubated with cell culture media and MTT reagent [3-(4,5-dimethylthiazol-2-yl)-2,5-diphenyltetrazolium bromide (5 mg/ml)] for 3 h, at which point media and MTT are removed and replaced with control solvent (DMSO) to solubilize the MTT product. The absorbance of the MTT product was then measured at 570 nm on a plate reader following 20 min incubation at room temperature. Annexin-V FITC and 7-AAD staining was performed according to manufacturer’s instructions (Invitrogen 88-8102-74). Stained cells were analyzed on a Thermo Scientific Attune NxT flow cytometer with a 488 nm laser, as described previously [44].

### Transmission electron microscopy (TEM)

TEM imaging was performed according to a protocol described previously [44]. Briefly, PND10 hearts were fixed (3% glutaraldehyde in PBS, pH 7.4) for 3 h at room temperature. Hearts were treated with a post-fixation step using 1% osmium tetroxide in phosphate buffer for 2 h at room temperature, followed by an alcohol dehydration series before embedding in Epon. TEM was performed with a Philips CM10, at 80 kV, on ultra-thin sections (100 nm on 200 mesh grids). Hearts were stained with uranyl acetate and counterstained with lead citrate.

### Immunoblotting

Protein isolation and quantification was performed as described previously [4]. Extracts were resolved via SDS-PAGE and later transferred to a PVDF membrane using an overnight transfer protocol. Immunoblotting was carried out using primary antibodies in 5% powdered milk or BSA (as per the manufacturer’s instructions) dissolved in TBST. Horseradish peroxidase-conjugated secondary antibodies (Jackson ImmunoResearch Laboratories; 1:5000) were used in combination with enhanced chemiluminescence (ECL) to visualize bands. The following antibodies were used: HMGB1 (CST # 3935), Rodent-specific Bnip3 (CST # 3769), Myc-Tag (CST # 2272), HA-Tag (CST # 3724), AIF (CST # 5318), MEK1/2 (CST # 8727), SERCA (Sigma MA3-919), DRP1 (CST # 8570), phospho-DRP1 [(ser616), (CST # 3455)], OPA1 (CST # 80471), Actin (sc-1616), and Tubulin (CST #86298). For detection of phosphorylation of Bnip3 at threonine-181, a custom rabbit polyclonal antibody was generated by Abgent using the following peptide sequence: AIGLGIYIGRRLp(T)TSTSTF.

### Real-time PCR

Total RNA was extracted from pulverized frozen tissue or from cultured cells by TRIzol method. cDNA was generated using SuperScript IV VILO Master Mix with ezDNase (Thermo #11766050) and q-RT-PCR performed using TaqMan Fast Advanced master mix (Thermo #4444965) on a CFX384 Real-Time PCR Instrument. The primers used were provided through ThermoFisher custom plating arrays (see Supplement 1 and 2 for assay list).

### Cardiac and cellular lactate, ATP and extracellular acidification

Cardiac lactate was quantified using the bioluminescent Lactate-Glo™ Assay (Promega #J5021) in deproteinized PND10 heart samples, as per the manufacturer’s protocol. Luminescence was detected and quantified using a Fluostar Optima microplate reader (BMG Labtech, Ortenberg, Germany). Cardiac and H9c2 ATP content was determined using a the Adenosine 5′-triphosphate (ATP) Bioluminescent Assay Kit (Sigma #FLAA-1KT), and normalized to DNA content as described previously [45]. Extracellular acidification and oxygen consumption was determined on a Seahorse XF-24 Extracellular Flux Analyzer in combination with Seahorse Mitochondrial Stress Test with drug concentrations as follows: 1 uM Oligomycin, 2 μM FCCP and 1 μM rotanone/antimycin A (Agilent Seahorse #1030f15-100). Calculated oxygen consumption rates were determined as per the manufacturer’s instructions (Mitochondrial Stress Test; Seahorse).

### Mitochondrial swelling and CRC assays

Mitochondrial swelling and calcium retention capacity (CRC) assays were performed using a cuvette–based fluorometric system (Horiba Scientific) which allows for the simultaneous detection of both fluorescence and absorbance, as described previously [46]. Hearts were minced in mitochondrial isolation buffer and homogenized using a 2 mL Teflon-glass homogenizer. Mitochondria were enriched by differential centrifugation at 4 °C. The mitochondrial isolation buffer consisted of 225 mM mannitol, 75 mM sucrose, 5 mM HEPES, and 1 mM EGTA, pH 7.4. Within the cuvette, 2 mg of mitochondria were incubated with 250 nM Calcium Green-5N (Invitrogen), 7 mM pyruvate (Sigma-Aldrich), and 1 mM malate (Sigma-Aldrich) in 1 ml of hypotonic KCl buffer (125 mM KCl, 20 mM HEPES, 2 mM KH2PO4, 40 μM EGTA, pH 7.2). In some experiments, mitochondria were incubated with 10 μM misoprostol for 5 min prior to the start of the assay. Mitochondria were then pulsed with sequential additions of CaCl2 (20 μM) over specific increments of time until mitochondrial swelling occurred.

### Phospho peptide mapping

Synthetic peptides (GeneScript) were resuspended at a concentration of 1 mg/ml. These peptides were used as the substrate in a PKA kinase assay kit (New England Biolabs, #P6000S) according to the manufacturer’s instructions, with the exception that [32 P]-ATP was replaced with fresh molecular biology grade ATP. The Kemptide substrate (Enzo Life Sciences; #P-107; LRRASLG) was used as a positive control in each assay. Before mass spectrometry analysis, kinase assays were prepared using C18 ZipTips (EMD Millipore, Etobicoke, ON, Canada). Samples in 50% acetonitrile and 0.1% formic acid were introduced into a linear ion-trap mass spectrometer (LTQ XL: ThermoFisher, San Jose, CA, USA) via static nanoflow, using a glass capillary emitter (PicoTip: New Objective, Woburn, MA, USA), as described previously [47].

### Statistics

Data are presented as mean ± standard error (S.E.M.) from 3 independent cell culture experiments. Differences between groups in imaging experiments with only 2 conditions were analyzed using an unpaired two-sided t-test, where (*) indicates *P* < 0.05 compared with control. Experiments with 4 or more conditions were analyzed using a 1-way ANOVA, or 2-way ANOVA where appropriate, with subsequent Tukey test for multiple comparisons, where (*) indicates *P* < 0.05 compared with control, and (**) indicates *P* < 0.05 compared with treatment. All statistical analysis was done using GraphPad Prism software. Exact N values are provided within the figure legends.

## Results

### Misoprostol prevents hypoxia-induced contractile and mitochondrial dysfunction in vivo

**Fig. 1:**
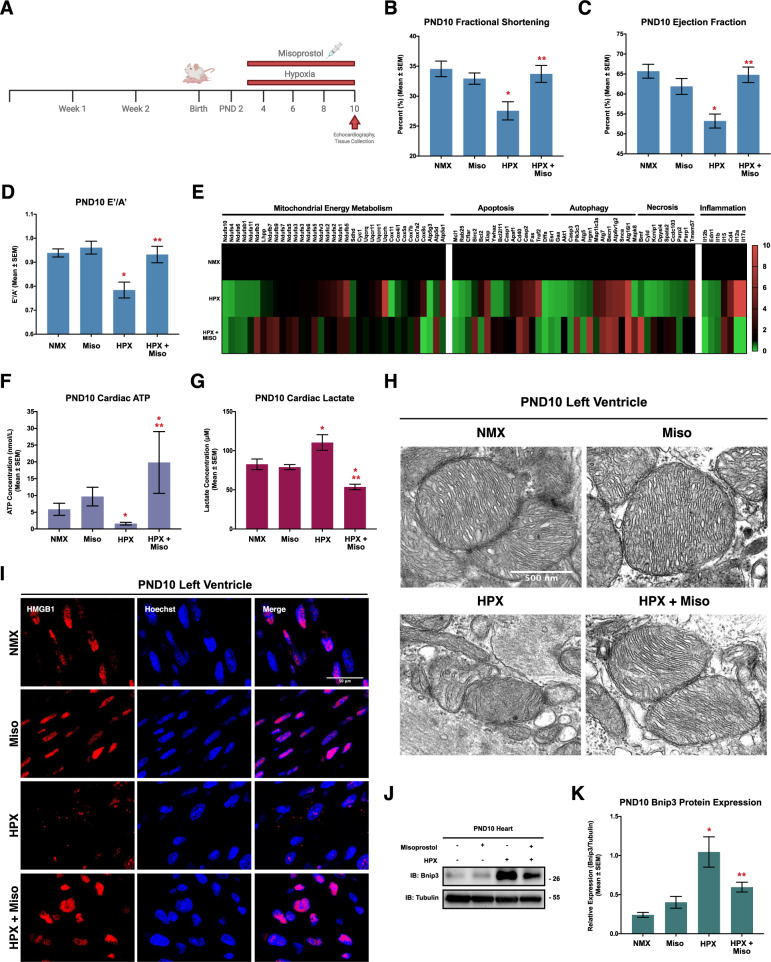
Misoprostol prevents hypoxia-induced contractile and mitochondrial dysfunction in vivo. **A** Schematic of the mouse model of neonatal hypoxia, where mice are exposed to hypoxia (10% O_2_) with or without 10 μg/kg misoprostol daily from PND3-10. **B** Fractional shortening, (**C**) Ejection fraction, and (**D**) E’/A’ ratio, for 4-6 post-natal day (PND10) mice treated as in (A), as determined by transthoracic echocardiography. **E** PCR-based array performed on RNA isolated from PND10 mouse ventricles (*n* = 3 animals per group) treated as in (**A**), where green indicates a downregulation of expression (<1), and red indicates an upregulation of expression (>1), relative to the normoxic control (1). **F** Measurement of ATP content in PND10 mouse ventricles (*n* = 6–8 animals per condition) treated as in (**A**). **G** Measurement of cardiac lactate content in the PND10 mouse ventricle (*n* = 6–8 animals per condition) treated as in (**A**). **H** PND10 hearts treated as in (**A**) and imaged via transmission electron microscopy. Images showing mitochondrial morphology. **I** PND10 hearts treated as in (**A**) and stained with DAPI (Blue) and probed for high mobility group box 1 (HMGB1, red). Hearts were imaged via confocal microscopy. **J** Representative immunoblot of heart protein extracts from post-natal day (PND10) mice treated as in (**A**). Extracts were immunoblotted as indicated. **K** Relative Bnip3 gene expression from the PND10 mouse ventricles of animals (*n* = 6–9 animals per group) treated as in (**A**). All data are represented as mean ± S.E.M. *P < 0.05 compared with control, while **P < 0.05 compared with hypoxia treatment, determined by 1-way ANOVA or 2-way ANOVA where appropriate. Three * indicates *P* < 0.05 compared to both control and treatment conditions.

Given that cardiac dysfunction has been implicated in neonatal hypoxic injury, we performed echocardiography in mice exposed to 10% oxygen from PND 3–10 (Fig. 1A) [4, 48]. This revealed significant contractile dysfunction in hypoxic mice, including reductions in fractional shortening (FS), ejection fraction (EF), and alterations in left ventricle filling (E’/A’) (Fig. 1B–D). However, when hypoxic mice were treated with misoprostol, contractility and filling of the heart returned to control levels (Fig. 1B–D).

We also performed PCR arrays to assess the expression of genes associated with mitochondrial metabolism, cell death pathways, and inflammatory cytokines (Fig. 1E; Supplementary 1–2). We observed changes in mRNA associated with the mitochondrial electron transport chain (ETC), including of NADH ubiquinone oxidoreductase and ATP synthase; however, very few of these genes were returned to control levels with misoprostol treatment. Interestingly, interleukins (IL) 17a and 12a, involved in innate- and T cell-mediated inflammation, were increased during hypoxia and reduced below detectable levels by misoprostol (Fig. 1E).

Next, we determined if the hypoxic neonatal heart demonstrated signs of mitochondrial dysfunction. We assessed cardiac ATP and lactate content and observed a significant reduction in cardiac ATP along with a corresponding increase in lactate (Fig. 1F, G). Importantly, misoprostol treatment reduced lactate content, whereas ATP levels were elevated beyond that observed in the normoxic animals. We then used transmission electron microscopy (TEM) to visualize mitochondrial morphology, which revealed that hypoxia altered mitochondrial structure; however, these changes were prevented when hypoxic animals were treated with misoprostol (Fig. 1H).

Based on the changes in mRNAs involved necrosis and inflammation, we examined the subcellular distribution of HMGB1, a nuclear protein that is released into the interstitium during necrosis where it acts as an alarmin or damage-associated molecular pattern (DAMP) to engage an inflammatory response. Using confocal immunofluorescence, hypoxia elicited a marked decrease in HMGB1 nuclear localization; however, when combined with misoprostol treatment, HMGB1 remained in the nucleus (Fig. 1I). Given the central role of Bnip3 in hypoxia-induced necrosis, we also assessed its expression by western blot, and observed that Bnip3 expression was increased by hypoxia, and partially reduced by misoprostol treatment (Fig. 1J, K).

These results indicate that hypoxia leads to cardiac bioenergetic collapse resulting in alterations to contractile function and a necroinflammatory phenotype. Treatment with misoprostol at least transiently maintains mitochondrial function, despite changes in mitochondrial gene expression, while preventing the necroinflammatory and contractile dysfunction. Finally, although misoprostol can repress Bnip3 expression in other tissues, it only partially reduced Bnip3 in the hypoxic neonatal heart, suggesting that additional pharmacodynamic mechanisms operate in this organ.

### Misoprostol prevents hypoxia-induced mitochondrial dysfunction in rodent and human cardiomyocytes

**Fig. 2:**
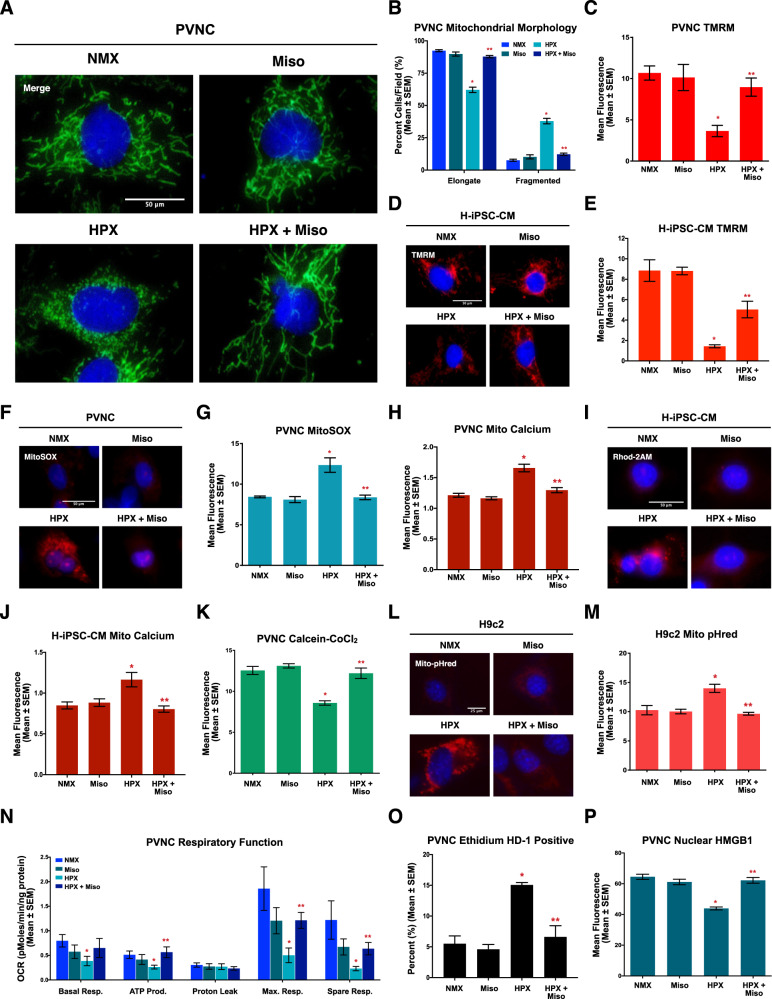
Misoprostol prevents hypoxia-induced mitochondrial perturbations in primary neonatal cardiomyocytes. **A** Primary ventricular neonatal cardiomyocytes (PVNCs) treated with 10 μM misoprostol (Miso) with or without exposure to 1% O_2_ (HPX) for 24 h. MitoView Green was included in all conditions to show mitochondrial morphology. Cells were stained with hoechst (blue) and imaged by standard fluorescence microscopy. **B** Quantification of cells in (**A**), where the number of cells with elongated and fragmented mitochondria are expressed as a percentage of all transfected cells in 30 random fields, across 3 independent experiments. **C** Quantification of PVNC’s treated as in (**A**). Cells were stained with TMRM (red) and hoechst (blue) and imaged by standard fluorescence microscopy. Red fluorescent signal was normalized to cell area and quantified in 30 random fields, across 3 independent experiments. **D** Human induced pluripotent stem cell-derived cardiomyocytes (H-iPSC-CMs) treated as in (**A**). Cells were stained with TMRM (red) and hoechst (blue) and imaged by standard fluorescence microscopy. **E** Quantification of cells in (**D**), as in (**C**). **F** PVNC’s treated as in (**A**). Cells were stained with MitoSOX (Red) and hoechst (blue) and imaged by standard fluorescence microscopy. **G** Quantification of cells in (**F**), as in (**C**). **H** Quantification of PVNC’s treated as in (**A**). Cells were stained with dihydrorhod-2AM (Red) and hoechst (blue) and imaged by standard fluorescence microscopy and quantified as in (**C**). **I** H-iPSC-CMs treated as in (**A**). Cells were stained with dihyrorhod-2AM (Red) and hoechst (blue) and imaged by standard fluorescence microscopy. **J** Quantification of cells in (**I**), as in (**C**). **K** Quantification of PVNC’s treated as in (**A**). Cells were stained with hoechst (blue) and calcein-AM quenched by cobalt chloride (CoCl_2_, 5 μM) to assess permeability transition, where green fluorescent signal was normalized to cell area and quantified in 30 random fields, across 3 independent experiments. **L** H9c2 cells treated as in (**A**), GW-1-Mito-pHred (red) was included in all conditions to visualize mitophagy. Cells were stained with hoechst (blue) and imaged by standard fluorescence microscopy. **M** Quantification of cells in (**L**), where red fluorescent signal was normalized to cell area and quantified in 15 random fields, across 3 independent experiments. **N** Calculated oxygen consumption rates (OCR) determined by Seahorse XF-24 analysis to evaluate mitochondrial function. **O** Quantification of PVNC’s treated as in (**A**). Live cells were stained with calcein-AM (green), and necrotic cells were stained with ethidium homodimer-1 (red). Percent (%) dead was calculated across 30 random fields, across 3 independent experiments. **P** Quantification of PVNC’s treated as in (**A**). Cells were fixed, stained with hoechst (blue), and immunofluorescence was performed using a HMGB1 primary antibody (green). Cells were then imaged by standard fluorescence microscopy. Green fluorescent signal was then normalized to nuclear area and quantified in 30 random fields, across 3 independent experiments. All data are represented as mean ± S.E.M. *P < 0.05 compared with control, while **P < 0.05 compared with hypoxia treatment, determined by 1-way ANOVA or 2-way ANOVA where appropriate. Three * indicates *P* < 0.05 compared to both control and hypoxia treatment.

To investigate how misoprostol regulates mitochondrial function, we employed primary ventricular neonatal cardiomyocytes (PVNCs). When exposed to hypoxia, we observed a significant increase in mitochondrial fragmentation (Fig. 2A, B). However, when PVNCs were concurrently treated with misoprostol, mitochondria returned to their normal networked appearance (Fig. 2A, B). Next, we used TMRM to assess mitochondrial membrane potential (ΔѰm), and observed that hypoxia significantly reduced ΔѰm, which was restored by misoprostol treatment (Fig. 2C). Further, we determined that this response was conserved in hypoxia-exposed human induced pluripotent stem cell (iPSC)-derived cardiomyocytes (H-iPSC-CMs)(Fig. 2D, E). We also assessed mitochondrial superoxide using MitoSOX staining, and observed increased superoxide production in hypoxic cells, which was abrogated in the presence of misoprostol (Fig. 2F, G).

To investigate how hypoxia alters subcellular calcium dynamics, we stained cardiomyocytes with a reduced form of Rhod-2AM (dihydrorhod2-AM), which provides specificity for mitochondrial calcium imaging. We observed that hypoxia significantly increased mitochondrial calcium content in both PVNCs and H-iPSC-CMs (Fig. 2H–J). However, when hypoxic cardiomyocytes were treated with misoprostol, mitochondrial calcium accumulation was prevented (Fig. 2H–J). We also assessed MPT in hypoxic cardiomyocytes using the calcein-CoCl_2_ method, where hypoxia resulted in a loss of mitochondrial puncta, indicative of permeability transition, while cells that were treated with misoprostol maintained mitochondrial staining (Fig. 2K). Using a mitophagy biosensor, called Mito-pHred, we observed that hypoxia significantly increased mitochondrial autophagy; however, this was also prevented by misoprostol (Fig. 2L, M).

We also examined the effect of misoprostol on mitochondrial respiration. As shown in Fig. 2N, hypoxia significantly reduced basal, maximal, and spare respiratory capacity, resulting in a reduction in the calculated mitochondrial ATP production. Consistent with our observations in vivo, misoprostol treatment abrogated this effect (Fig. 2N). Next, we determined if improved mitochondrial function, elicited by misoprostol, delayed cell death. We performed MTT assays and observed that hypoxia significantly reduced cell viability, which was attenuated in the presence of misoprostol (Supplement 3A). Next, we performed live/dead assays using ethidium homodimer-1 to mark the nuclei of cells with disrupted membrane integrity [49]. We observed that hypoxia significantly increased the percentage of red staining nuclei, which was prevented in myocytes treated with misoprostol (Fig. 2O). To confirm necrotic cell death, we also assessed HMGB1 subcellular distribution. While hypoxic PVNCs demonstrated a significant decrease in nuclear HMGB1 immunofluorescence, the addition of misoprostol restored HMGB1 staining (Fig. 2P). Collectively, these results indicate that misoprostol prevents hypoxia-induced mitochondrial dysfunction, necrosis, and alarmin/DAMP release in neonatal cardiomyocytes.

### Misoprostol prevents Bnip3-induced mitochondrial dysfunction and cell death

**Fig. 3:**
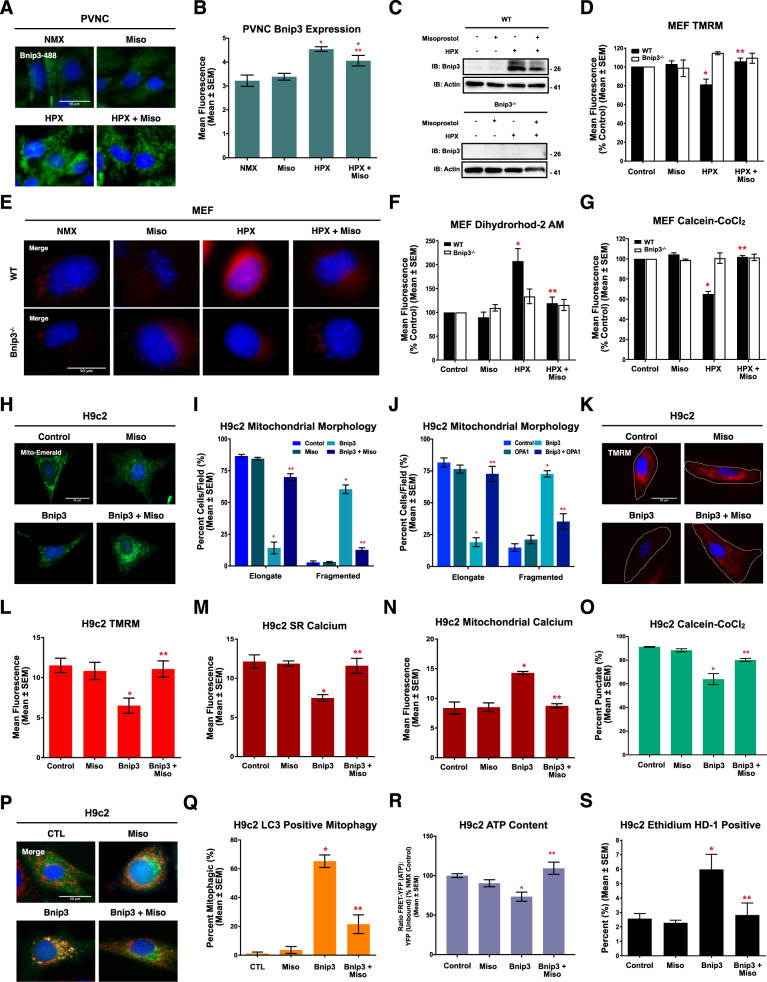
Misoprostol prevents Bnip3-induced mitochondrial perturbations and cell death in MEFs and H9c2 cells. **A** PVNC’s treated with 10 μM misoprostol (Miso) with or without exposure to 1% O_2_ (HPX) for 24 h. Cells were fixed, stained with hoechst (blue), and immunofluorescence was performed using a Bnip3 primary antibody (green). Cells were then imaged by standard fluorescence microscopy. **B** Quantification of cells in (**A**), where green fluorescent signal was normalized to cell area and quantified in 30 random fields, across 3 independent experiments. **C** Immunoblot for Bnip3 in protein extracts from WT and Bnip3^−/−^ MEFs treated as in (**A**). **D** Quantification of WT and Bnip3^−/−^ mouse embryonic fibroblasts (MEFs) treated as in (**A**). Cells were stained with TMRM (red) and hoechst (blue) and imaged by standard fluorescence microscopy. Red fluorescent signal was normalized to cell area and quantified in 15 random fields, across 3 independent experiments. **E** WT and Bnip3^−/−^ MEFs treated as in (**A**). Cells were stained with hoechst (blue) and dihydrorhod-2AM to stain mitochondrial calcium. **F** Quantification of (**E**) as in (**D**) in 15 random fields, across 3 independent experiments. **G** Quantification of WT and Bnip3^−/−^ MEFs treated as in (**A**). Cells were stained with hoechst (blue) and calcein-AM quenched by cobalt chloride (CoCl_2_, 5 μM) to assess permeability transition. Green fluorescent signal was normalized to cell area and quantified in 15 random fields, across 3 independent experiments. **H** H9c2 cells transfected with pcDNA3 (control) or Myc-Bnip3 and treated with 10 μM misoprostol (Miso) or PBS control for 16 h. Mito-Emerald (green) was included in all conditions to show transfected cells and mitochondrial morphology. Cells were stained with hoechst (blue) and imaged by standard fluorescence microscopy. **I** Quantification of cells in (H), where the number of cells with elongated and fragmented mitochondria are expressed as a percentage of all transfected cells in 30 random fields, across 3 independent experiments. **J** Quantification of H9c2 cells transfected with pcDNA3 (control), Myc-Bnip3 and/or myc-Opa1. Mito-Emerald (green) was included in all conditions as in (**H**) and cells were stained with hoechst (blue) and imaged by standard fluorescence microscopy. Quantification as in (**I**). **K** H9c2 cells treated as in (**H**). CMV-GFP (outline) was included in all conditions to indicate transfected cells Cells were stained and imaged as in (**D**). **L** Quantification of cells in (**K**) as in (**D**). **M** Quantification of H9c2 cells treated as in (**H**). ER-LAR-GECO (red) was included in all conditions to indicate ER calcium content. Cells were stained and imaged as in (**H**). Quantification was performed as in (**D**) in 30 random fields, across 3 independent experiments. **N** Quantification of H9c2 cells treated as in (**H**). Mito-CAR-GECO (red) was included in all conditions to indicate mitochondrial calcium content. Cells were stained and imaged as in (**D**). Quantification was performed as in (**D**) in 30 random fields, across 3 independent experiments. **O** Quantification of H9c2 cells treated as in (**H**) and CMV-ds-RED was included in all conditions to indicate transfected cells. Cells were stained, imaged and quantified as in (**G**) across 30 random fields, across 3 independent experiments. **P** H9c2 cells treated as in (**H**). LC3-GFP (green) was included in all conditions to show transfected cells and autophagic puncta. Cells were stained with hoechst (blue) and MitoTracker red (red) and imaged by standard fluorescence microscopy. **Q** Quantification of cells in (**P**), where the number of cells with LC3-GFP and MitoTracker co-localization are expressed as a percentage of all transfected cells in 10 random fields. **R** Quantification of H9c2’s treated as in (**H**). ATeam was used to indicate cytosolic ATP content. Cells were imaged by FRET-based microscopy. FRET-YFP (ATP) signal was divided by the YFP (unbound biosensor) signal in 15 random fields across 3 independent experiments. **S** Quantification of H9c2 cells treated as in (**H**). Live cells were stained with calcein-AM (green), and necrotic cells were stained with ethidium homodimer-1 (red) and are expressed as percent (%) dead in 30 random fields, across 3 independent experiments. All data are represented as mean ± S.E.M. *P < 0.05 compared with control, while **P < 0.05 compared with hypoxia or Bnip3 treatment, determined by 1-way ANOVA or 2-way ANOVA where appropriate. Three * indicates *P* < 0.05 compared to both control and treatment conditions.

Using PVNCs and mouse embryonic fibroblasts (MEFs), we observed that hypoxia increased Bnip3 expression (Fig. 3A–C). Consistent with our previously results, misoprostol treatment reduced Bnip3 expression; however, it still remained elevated relative to control cells [4]. To determine the necessity of Bnip3 during hypoxia-induced mitochondrial dysfunction, we used MEFs isolated from Bnip3^−/−^ embryos, described previously [6, 31]. TMRM analysis revealed that hypoxic WT MEFs displayed a significant reduction in ΔѰm, which was prevented in the Bnip3^−/−^ MEFs, or misoprostol treatment of WT MEFs (Fig. 3D). Hypoxia also increased mitochondrial calcium in the WT MEFs, but not in the Bnip3^−/−^ MEFs, while misoprostol provided protection against mitochondrial calcium accumulation in the WT cells (Fig. 3E, F). In addition, Bnip3^−/−^ MEFs were less susceptible to hypoxia-induced MPT, while misoprostol protected WT MEFs (Fig. 3G).

Next, we used H9c2 cardiomyoblasts to determine if misoprostol could inhibit Bnip3 function. Using mito-Emerald to visualize mitochondrial morphology (Fig. 3H, I), Bnip3 expression resulted in a fragmented mitochondrial appearance. However, when Bnip3-expressing cells were treated with misoprostol, mitochondria retained a networked appearance (Fig. 3H, I). Given previous reports of Bnip3 regulating mitochondrial morphology through an interaction with Opa1, we also tested if overexpression of Opa1 rescues Bnip3-induced mitochondrial fragmentation. Shown in Fig. 3J, Bnip3 induced mitochondrial fragmentation was prevented with the co-expression of Opa1. We also evaluated phosphorylated DRP1 and Opa1 expression in Bnip3-expressing H9c2 cells, and observed no impact on these targets (Supplementary 3B, C). Using TMRM we observed that Bnip3 expression induced mitochondrial depolarization, which was restored in the presence of misoprostol (Fig. 3K, L).

We next investigated the role of subcellular calcium as a mechanism of Bnip3-induced mitochondrial dysfunction, using organelle-targeted calcium biosensors called GECOs. When expressed with the ER-targeted biosensors (ER-LAR-GECO), Bnip3 significantly reduced ER calcium stores (Fig. 3M). Concurrently, using the mitochondrial targeted biosensor (Mito-CAR-GECO), Bnip3 increased mitochondrial calcium (Fig. 3N), suggesting a shift of calcium from the ER to the mitochondria. Furthermore, this shift in calcium was prevented by misoprostol (Fig. 3M, N). We also observed that Bnip3 expression caused MPT, which was opposed by misoprostol (Fig. 3O). Additionally, we assessed mitophagy by assessing the colocalization of LC3 with mitochondria in cells expressing Bnip3 and observed punctate colocalization, suggesting enhanced mitophagy. However, this observation was absent with the addition of misoprostol (Fig. 3P, Q).

Next, we determined if Bnip3-induced mitochondrial dysfunction led to a depletion of cellular ATP. Using the ATeam biosensor [39], we observed that ATP content was significantly reduced in Bnip3-expressing H9c2 cells and that this effect was prevented by misoprostol (Fig. 3R). In addition, Bnip3 increased in the number dead cells per field in a live/dead assay, which was also prevented with misoprostol (Fig. 3S). We also performed Annexin V/7-AAD staining and flow cytometry analysis, and observed that Bnip3 enhanced necrotic cell death (7-AAD staining), but had little impact on Annexin V staining (Supplement 3D). Furthermore, treatment with misoprostol attenuated the increases in 7-AAD staining induced by Bnip3 (Supplement 3D). Together this data indicates that misoprostol is capable of inhibiting Bnip3 function and preventing mitochondrial perturbations associated with necrosis.

### Misoprostol modulates a novel PKA phosphorylation site on Bnip3 at Thr-181

**Fig. 4:**
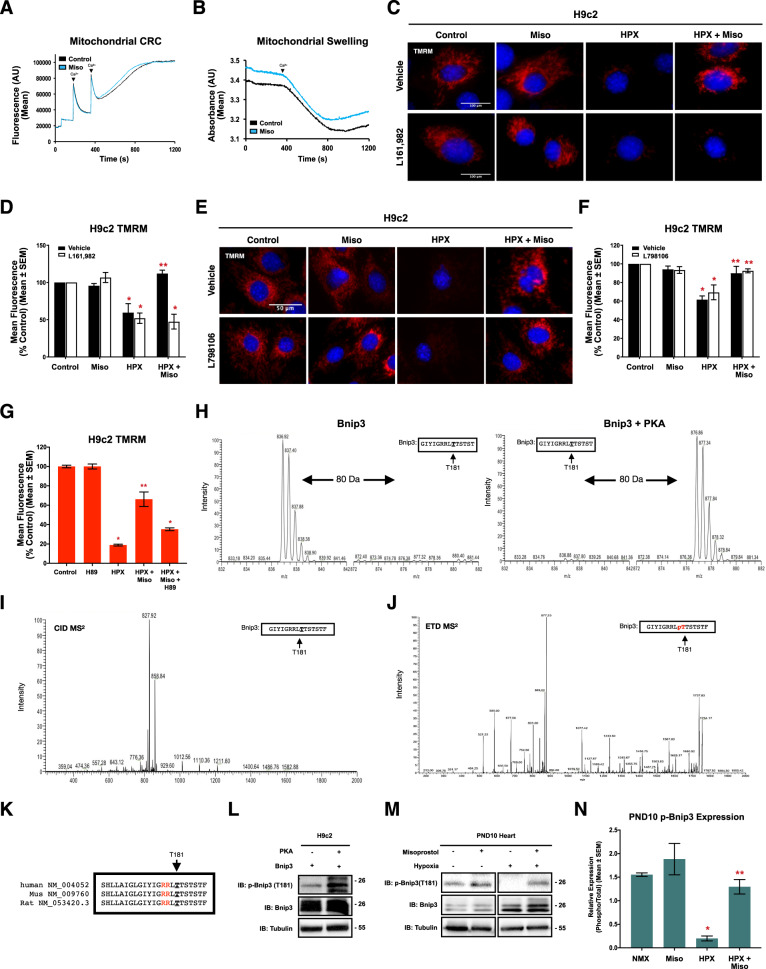
Misoprostol stimulates EP4 receptors to activate PKA and promote the modulation of a novel PKA phosphorylation site on Bnip3 at Thr-181 in cells and the PND10 neonatal heart. **A** Mitochondrial calcium retention capacity (CRC) in isolated mitochondria exposed to 10 μM misoprostol (Miso) or PBS control. **B** Mitochondrial swelling assay in isolated mitochondria treated as in (**A**). **C** H9c2 cells exposed to 1% O_2_ (HPX) or 21% O_2_ (control) and treated with 10 μM misoprostol (Miso) or PBS control for 24 h. 10 μM L161,982 was also included in half of the conditions. Cells were stained with TMRM (red) and hoechst (blue) and imaged by standard fluorescence microscopy. **D** Quantification of cells in (**C**), where red fluorescent signal was normalized to cell area and quantified in 30 random fields, across 3 independent experiments. **E** H9c2 cells treated, stained and imaged as in (**C**). 1 μM L798106 was also included in half of the conditions. **F** Quantification of cells € (**E**), as in (**D**). **G** Quantification of H9c2 cells treated as in (**C**) with the addition of 10 μM H-89 for 24 h. Cells were imaged as in (**C**) and quantified as in (**D**). **H** SIM scan of the wild-type peptide spanning the PKA site of Bnip3. The unphosphorylated peptide has a 837 m/z (z = 2^+^) (Left), putative phosphorylation showing an increased m/z of 40 that corresponds to PO_3_ (M = 80.00 Da) (Right). **I** MS^2^ spectra following collision induced dissociation (CID) of the mass shifted ion from (**H**) yielding a product-ion consistent with a neutral loss of H_3_PO_4_. **J** MS^2^ spectra following electron transfer dissociation (ETD) of a triply charged mass-shifted ion following kinase reaction (not shown). Analysis of this fragmentation spectra confirmed that threonine-181 is the preferred phosphorylation residue. **K** Alignment of evolutionarily conserved T181 phosphorylation site (bold and underlined), and 14-3-3 binding motifs (Red) for Bnip3 in human, mouse and rat. **L** Immunoblot of H9c2 cells transfected with Myc-Bnip3 and/or PKA for 16 h. **M** Representative immunoblot of heart protein extracts from post-natal day (PND10) mice exposed to hypoxia (10% O_2_) ± 10 μg/kg misoprostol from PND3-10. Extracts were immunoblotted as indicated. **N** Phospho-Bnip3 densitometry for extracts in (**M**), representing an N of 3 male PND10 mouse hearts. All data are represented as mean ± S.E.M. *P < 0.05 compared with control, while **P < 0.05 compared with hypoxia treatment, determined by 1-way ANOVA or 2-way ANOVA where appropriate.

We sought to determine if misoprostol was acting directly on the mitochondria or if a plasma membrane mediator was involved. Thus, we isolated cardiac mitochondria and treated directly with misoprostol or vehicle, followed by mitochondrial calcium retention capacity (CRC) and mitochondrial swelling assays. Shown in Fig. 4A, B, misoprostol had no direct effect on isolated mitochondria treated with exogenous calcium.

Next, we investigated the role of prostaglandin cell surface receptors. Monitoring ΔѰm, we applied L161,982, an EP_4_ receptor antagonist, in combination with hypoxia and misoprostol treatments. Misoprostol treatment prevented hypoxia-induced mitochondrial depolarization, but this rescue was lost when the EP_4_ receptor was inhibited (Fig. 4C, D). Conversely, EP_3_ receptor antagonism with L798106 had no effect (Fig. 4E, F). In addition, we expressed a PKA biosensor and treated with misoprostol, which demonstrated peak activation 30 min following misoprostol treatment (Supplementary 4A). These results suggest that EP_4_ activation of PKA might be a mechanism by which misoprostol prevents MPT. To explore the role of PKA in misoprostol-induced protection, we used H89, a PKA inhibitor, in combination with hypoxia and misoprostol treatments. Hypoxia exposure reduced ΔѰm, which was restored with misoprostol; however, when combined with H89, this restoration was lost (Fig. 4G).

To investigate if PKA can directly phosphorylate Bnip3, we analyzed the mouse Bnip3 amino acid sequence, and identified conserved PKA phosphorylation motifs at serine (Ser)-107 and threonine (Thr)-181. We engineered peptides spanning these regions and performed in vitro kinase reaction with purified PKA, followed by mass spectrometry analysis. For the peptides spanning Ser-107, no discernible peaks corresponding to phosphorylation were observed (Supplementary 4). However, for the peptides spanning Thr-181, single ion monitoring (SIM) displayed a predominant peak at m/z of 836.92 (z = 2^+^) for the control. Following incubation with PKA, the peptide m/z increased by 40 suggesting phosphorylation (m/z 876.86 z = 2^+^; mass = 80.00 Da) (Fig. 4H). We also evaluated if this peptide could be phosphorylated at more than one residue, but we did not detect an increased m/z of 80 (Supplement 4). Next, we analyzed the MS^2^ spectra produced by collision-induced dissociation (CID) of the mass-shifted peptide with m/z 876.86 (z = 2^+^). CID fragments phospho-residues resulting in the loss of H_3_PO_4_, and the generation of a product-ion with a mass less 98 Da (m/z = 49 for z = 2^+^). CID yielded a product-ion with m/z = 827.92 (delta = 48.94), also indicating phosphorylation (Fig. 4I). Next, we subjected the triply charged phospho-peptide (m/z = 585.27; not shown) to electron transfer dissociation (ETD). This technique breaks peptide bonds but retains side-chain phosphorylation’s to determine specific phospho-residues. Using the ETD MS^2^ spectra, Mascot software identified threonine-181 of Bnip3 as the phosphorylated residue (Fig. 4J).

To confirm that PKA phosphorylates Bnip3, we used a custom phospho-antibody targeted to Thr-181, and co-expressed the catalytic subunit of PKA and Bnip3 and observed a marked increase in phosphorylation (p-Bnip3) (Fig. 4L). We next exposed H9c2 cells and PVNCs to misoprostol and observed an increase in endogenous Bnip3 phosphorylation (Supplementary 4). Finally, to determine if Bnip3 phosphorylation is regulated in vivo, we performed western blots on from neonatal mice exposed to hypoxia and observed a significant reduction in p-Bnip3, which was restored when mice were treated with misoprostol (Fig. 4M, N). In addition, we evaluated Bnip3 phosphorylation in adult rodent hearts and observed a significant decrease in p-Bnip3 in the viable border zone following 4-weeks of coronary ligation. However, by 8 weeks post ligation p-Bnip3 returned to control levels (Supplementary 5A–C). These results implicate Bnip3 phosphorylation at Thr-181 as a regulated event in vivo.

### Misoprostol inhibits Bnip3 through Thr-181

**Fig. 5:**
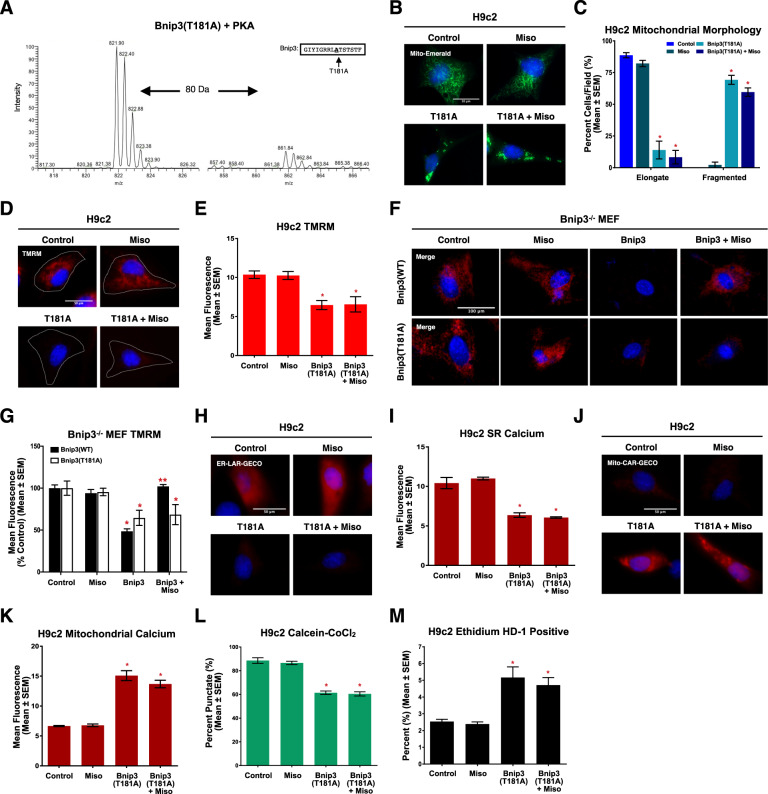
Misoprostol Inhibits Bnip3-induced mitochondrial perturbations and cell death through Thr-181 phosphorylation in H9c2 cells. **A** SIM scan of a mutated peptide where the PKA site at Threonine-181 is replaced with Alanine (left). On the right, phosphorylation of this mutate peptide is negligible at the predicted m/z that corresponds to the addition of a PO_3_ (M = 80.00 Da). **B** H9c2 cells transfected with pcDNA3 (control) or Myc-T181A and treated with 10 μM misoprostol (Miso) or PBS control for 16 h. Mito-Emerald (green) was included in all conditions to show transfected cells and mitochondrial morphology. Cells were stained with hoechst (blue) and imaged by standard fluorescence microscopy. **C** Quantification of cells in (**B**), where the number of cells with elongated and fragmented mitochondria are expressed as a percentage of all transfected cells in 30 random fields, across 3 independent experiments. **D** H9c2 cells treated as in (**B**). CMV-GFP (outline) was included in all conditions to indicate transfected cells Cells were stained with TMRM (red) and hoechst (blue) and imaged by standard fluorescence micros€y. **E** Quantification of cells in (**D**), where red fluorescent signal was normalized to cell area and quantified in 30 random fields, across 3 independent experiments. **F** Bnip3^−/−^ mouse embryonic fibroblasts (MEFs) treated as in (**B**) where either Myc-Bnip3(WT) or Myc-Bnip3(T181A) was transfected in Cells were stained with TMRM (red) and hoechst (blue) and imaged by standard fluorescence microscopy. **G** Quantification of cells in (**F**), where red fluorescent signal was normalized to cell area and quantified in 15 random fields, across 3 independent experiments. **H** H9c2 cells treated as in (**B**). ER-LAR-GECO (red) was included in all conditions to indicate ER calcium content. Cells were stained with hoechst (blue) and imaged by standard fluorescence microscopy. **I** Quantification of cells in (€as in (**E**) in 30 random fields, across 3 independent experiments. **J** H9c2 cells treated as in (**B**). Mito-CAR-GECO (red) was included in all conditions to indicate mitochondrial calcium content. Cells were stained with hoechst (blue) and imaged by standard fluorescence microscopy. **K** Quantification of cells i€J) as in (**E**) in 30 random fields, across 3 independent experiments. **L** Quantification of H9c2 cells treated as in (**B**) and CMV-ds.RED was included in all conditions to indicate transfected cells. Cells were stained with hoechst (blue) and calcein-AM quenched by cobalt chloride (CoCl_2_, 5 μM) to assess permeability transition. Quantification was done by calculating the percentage of cells with mitochondrial puncta in 30 random fields, across 3 independent experiments. **M** Quantification of H9c2 cells treated as in (B). Live cells were stained with calcein-AM (green), and necrotic cells were stained with ethidium homodimer-1 (red) and are expressed as percent (%) dead in 30 random fields, across 3 independent experiments. All data are represented as mean ± S.E.M. *P < 0.05 compared with control, while **P < 0.05 compared with Bnip3 treatment, determined by 1-way ANOVA or 2-way ANOVA where appropriate.

To understand the cellular role of Bnip3 phosphorylation, we first generated both a peptide and a plasmid replacing Thr-181 with an alanine residue (T181A). Using mass spectroscopy, we observed that the T181A peptide can no longer be phosphorylated (Fig. 5A). To demonstrate specificity, we engineered a peptide replacing the threonine at position-182 with an alanine and observed phosphorylation similar to the WT peptide (Supplementary 4). Next, we employed gain-of-function transfection studies with the Bnip3-T181A construct, in combination with mito-Emerald, to visualize mitochondrial morphology. Expression of T181A resulted in a fragmented mitochondrial phenotype; however, the T181A mutant was not inhibited by misoprostol treatment (Fig. 5B, C). In addition, misoprostol was not able to overcome the reduction in ΔѰm induced by T181A (Fig. 5D, E). To determine the necessity of Thr-181 as a down-stream target of misoprostol treatment, we reconstituted WT or T181A Bnip3 expression in Bnip3^−/−^ MEFs. We observed that both WT and T181A reduced ΔѰm; however, misoprostol treatment restored ΔѰm to control in the WT Bnip3 transfected cells but failed to restore ΔѰm in the presence of T181A (Fig. 5F, G).

When we investigated underlying calcium phenomena, we observed that T181A expression shifted calcium from the ER into the mitochondria. However, misoprostol was unable to prevent this calcium shift induced by T181A (Fig. 5H–K). Likewise, misoprostol was unable to prevent MPT and cell death elicited by the T181A mutant (Fig. 5L, M). These data demonstrate that phosphorylation of Bnip3 at Thr-181 is necessary for misoprostol to inhibit Bnip3 function.

### Thr-181 phosphorylation retains Bnip3 in the cytosol

**Fig. 6:**
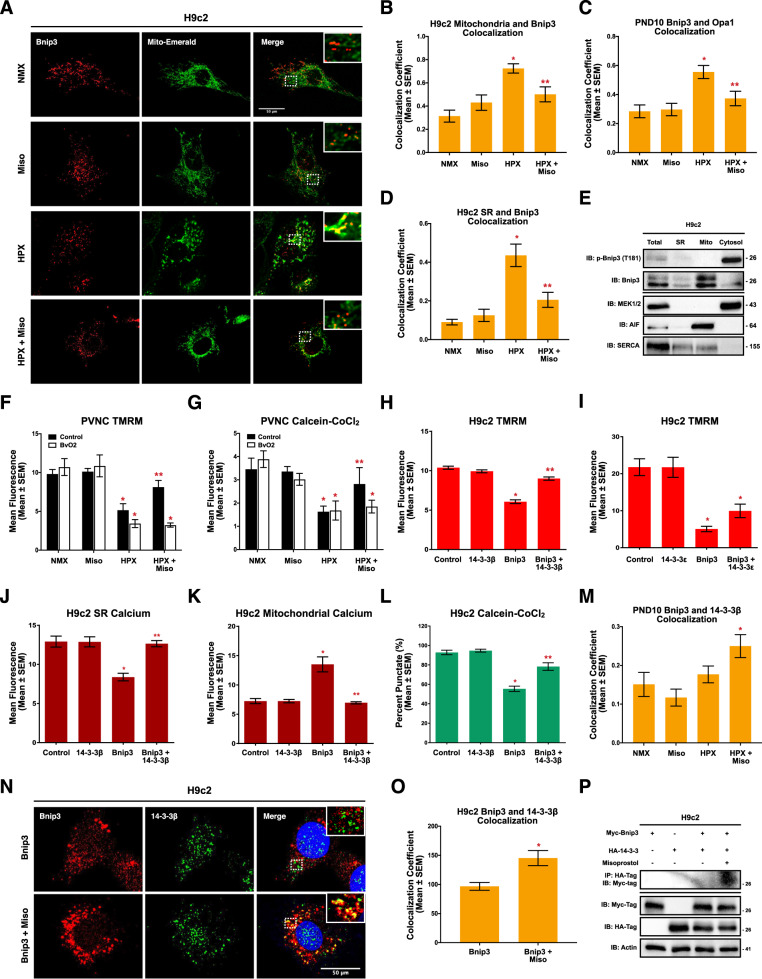
Bnip3 phosphorylation at Thr-181 retains Bnip3 in the cytosol through inhibitory interactions with 14-3-3β in H9c2 cells. **A** H9c2 cells treated with 10 μM misoprostol (Miso) ± 1% O_2_ (HPX) for 24 h. Myc-Bnip3 and Mito-Emerald (green) were included in each condition to visualize localization. Cells were fixed, stained with hoechst (blue), and immunofluorescence was performed using a Myc-tag primary antibody (Red). Cells were then imaged by standard confocal microscopy. **B** Quantification of cells in (**A**), where colocalization coefficient was calculated for 30 cells per condition across 10 random fields. **C** Quantification of immunofluorescence in PND10 hearts exposed to hypoxia (10% O_2_) ± 10 μg/kg misoprostol from PND3-10. Hearts were probed for Bnip3 (Red), Opa1 (Green), and stained with DAPI (Blue). Hearts were imaged by standard confocal microscopy and the colocalization coefficient was calculated in 20 fields per condition (*n* = 4 animals/conditions). **D** Quantification of H9c2 cells treated as in (**A**). ER-Emerald (green) was transfected in all conditions. Cells were fixed, stained with hoechst (blue), and immunofluorescence was performed using a Myc-tag primary antibody (Red). Cells were then imaged by standard confocal microscopy. Colocalization coefficient was calculated for 30 cells per condition across 10 random fields. **E** Fractionation of control treated H9c2. Protein extracts were fractionated and immunoblotted, as indicated. **F** Quantification of primary ventricular neonatal cardiomyocytes (PVNCs) treated with 10 μM misoprostol (Miso) ± 1% O_2_ (HPX) for 24 h. 5 μM BvO2 was included in half of the conditions to inhibit 14-3-3 protein activity. Cells were stained with TMRM (red) and hoechst (blue) and imaged by standard fluorescence microscopy. Red fluorescent signal was normalized to cell area and quantified in 20 random fields, across 2 independent experiments. **G** Quantification of PVNCs treated as in (**E**). Cells were stained with hoechst (blue) and calcein-AM quenched by cobalt chloride (CoCl_2_, 5 μM) to assess permeability transition, and imaged by standard fluorescence microscopy. The percentage of cells with mitochondrial puncta was calculated in 20 random fields, across 2 independent experiments. **H** Quantification of H9c2 cells transfected with pcDNA3 (control) or Myc-Bnip3 with and without HA-14-3-3β. CMV-GFP (outline) was included in all conditions to indicate transfected cells Cells were stained with TMRM (red) and hoechst (blue) and imaged by standard fluorescence microscopy. Red fluorescent signal was normalized to cell area and quantified in 30 random fields, across 3 independent experiments. **I** Quantification of H9c2s treated as in (**G**), with and without 14-3-3ε. Cells were stained and imaged as in (**H**) and quantified as an (**I**) across 30 random fields, in 3 independent experiments. **J** Quantification of H9c2’s treated as in (**H**). ER-LAR-GECO (red) was included in all conditions to indicate ER calcium content. Cells were stained and imaged as in (**H**). Red fluorescent signal was normalized to cell are in 30 random fields, across 3 independent experiments. **(K)** Quantification of H9c2’s treated as in (**H**). Mito-CAR-GECO (red) was included in all conditions to indicate mitochondrial calcium content. Quantification performed as in (**J**) in 30 random fields, across 3 independent experiments. **L** Quantification of H9c2’s treated as in (**H**), and CMV-ds.RED was included in all conditions to indicate transfected cells. Cells were stained with hoechst (blue) and calcein-AM quenched by cobalt chloride (CoCl_2_, 5 μM) to assess permeability transition. Where the percentage of cells with mitochondrial puncta was calculated in 30 random fields, across 3 independent experiments. **M** Quantification of immunofluorescence in PND10 hearts exposed to hypoxia (10% O_2_) ± 10 μg/kg misoprostol from PND3-10. Hearts were probed for Bnip3 (Red), 14-3-3β (Green), and stained with DAPI (Blue). Hearts were imaged by standard confocal microscopy and the colocalization coefficient was calculated in 20 fields per condition (*n* = 4 animals/conditions). **N** H9c2 cells transfected with Myc-Bnip3 ± 10 μM misoprostol (Miso) 18 h. Cells were fixed, stained with hoechst (blue), and immunofluorescence was performed using a Myc-tag (Red), and 14-3-3β (Green). Cells were then imaged by standard confocal microscopy. **O** Quantification of cells in (**N**), where colocalization coefficient was calculated for 30 cells per condition across 10 random fields. **P** Co-immunoprecipitation of HCT-116 cells expressing HA-14-3-3 and Myc-Bnip3. Proteins were pulled down with Myc and probed for HA-tag. Immunoblot was probed as indicated. All data are represented as mean ± S.E.M. *P < 0.05 compared with control, while **P < 0.05 compared with hypoxia or Bnip3 treatment, determined by 1-way ANOVA or 2-way ANOVA where appropriate.

To determine how phosphorylation at Thr-181 inhibits Bnip3 function, we expressed matrix-targeted mito-emerald, and performed confocal immunofluorescence for Bnip3 following exposure to hypoxia and/or misoprostol. As shown in Fig. 6A, B, we observed very little interaction between mito-emerald and Bnip3; however, when H9c2 cells were exposed to hypoxia the colocalization coefficient increased. Interestingly, we observed that this colocalization was abrogated by misoprostol (Fig. 6A, B). Using a similar approach, we assessed the colocalization between Bnip3 and Opa1 in the neonatal heart. At baseline there was very little interaction between the two proteins, which was significantly increased by hypoxia, but disrupted by misoprostol (Fig. 6C, Supplementary 6A). Similar results were obtained using an ER/SR-targeted emerald (Fig. 6D). Next, we determined the subcellular localization of p-Bnip3 through fractionation studies. We observed that Bnip3 is predominantly localized to the mitochondria, and to a lesser extent at the ER, while p-Bnip3 is cytosolic (Fig. 6E).

Analysis of Bnip3 sequence predicted that Thr-181 lies within a 14-3-3 domain. This family interacts with a RxxpTx motif (Bnip3: RRLpTT), which are commonly found within PKA and CaMKII sites (See Fig. 4K for alignment). As we recently determined that PKA-dependent phosphorylation of Nix (Bnip3L) increases its interaction with 14-3-3β [43], we investigated the role of these chaperones as a mechanism by which misoprostol inhibits Bnip3. Using hypoxia and misoprostol exposed PVNCs, we applied BvO2, a pan-14-3-3 inhibitor, and assessed ΔѰm and MPT. We observed that misoprostol’s ability to rescue of ΔѰm and MPT was prevented by BvO2 (Fig. 6F, G).

Next, we expressed Bnip3 and 14-3-3β, alone and in combination, in H9c2 cells. TMRM staining revealed that 14-3-3β was able to rescue Bnip3-induced mitochondrial depolarization (Fig. 6H). Similar experiments were conducted using 14-3-3ε, which was unable to restore ΔѰm, demonstrating some degree of isoform specificity (Fig. 6I). We also observed that 14-3-3β expression is sufficient to prevent the ER calcium depletion and mitochondrial calcium accumulation triggered by Bnip3 expression (Fig. 6J, K), and prevent MPT (Fig. 6L).

To determine if 14-3-3β and Bnip3 physical interact, we performed immunofluorescence targeting both Bnip3 and 14-3-3β following exposure to hypoxia and/or misoprostol in the neonatal heart. Although we observed little colocalization at baseline, possibly due to low expression of Bnip3 in the normoxic conditions, the combination of hypoxia and misoprostol treatment increased the colocalization coefficient between Bnip3 and 14-3-3β (Fig. 6M, Supplement 6B). To overcome the challenges associated with altered Bnip3 expression between normoxic and hypoxic conditions, we overexpressed both Bnip3 and 14-3-3β in H9c2 cells and performed confocal immunofluorescence. As shown in Fig. 6N and O, misoprostol was sufficient to increase colocalization. Next, we co-expressed HA-14-3-3β and myc-Bnip3 in H9c2 cells that were concurrently treated with misoprostol, and performed immunoprecipitation with an HA antibody. We detected myc-Bnip3 from the immunoprecipitation when cells were treated with misoprostol (Fig. 6P). Finally, we tested whether 14-3-3β expression could inhibit Bnip3 T181A function. We expressed T181A with and without 14-3-3β and assessed TMRM and Mito-CAR-GECO (Supplement 7A–D). Interestingly, 14-3-3β was ineffective at inhibiting the Bnip3 T181A mutant. Collectively, these data indicate that misoprostol promotes p-Bnip3 trafficking away from the mitochondria and ER through an interaction with 14-3-3β.

### Bnip3 ablation prevents hypoxia-induced contractile dysfunction and necroinflammation in the neonatal heart

**Fig. 7:**
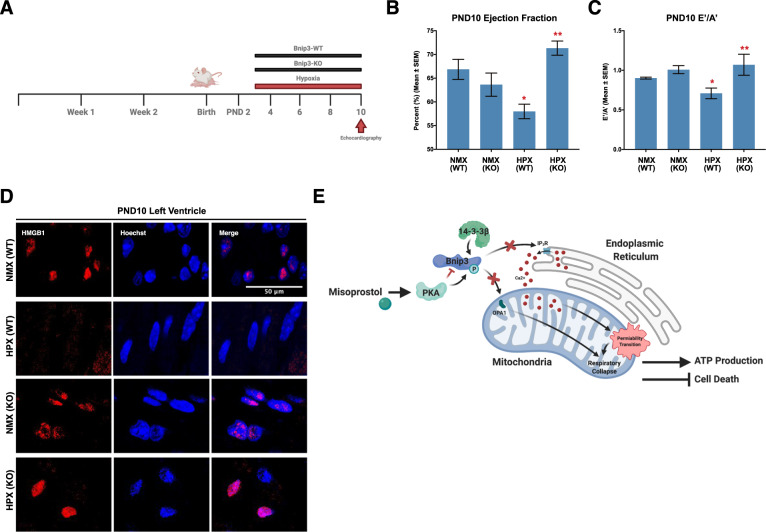
Bnip3 ablation prevents hypoxia-induced contractile dysfunction and necroinflammation in the PND10 neonatal heart. **A** Post-natal day 2 (PND2) Bnip3-WT and Bnip3-null mice are exposed to hypoxia (10% O_2_) from PND3-10, hearts were imaged and collected on PND10. **B** Ejection fraction and (**C**) E’/A’ ratio for PND10 animals treated as in (**A**), in 3-5 mice per group, as determined by transthoracic echocardiography. **D** PND10 hearts treated as in (**A**) and stained with DAPI (Blue) and probed for high mobility group box 1 (HMGB1, red). Hearts were imaged via €focal microscopy. **E** Proposed mechanism by which misoprostol inhibits Bnip3 at the mitochondria and ER to prevent necrotic cell death and necroinflammation. All data are represented as mean ± S.E.M. *P < 0.05 compared with WT control, while **P < 0.05 compared with WT hypoxia treatment, determined by 1-way ANOVA.

To determine if a direct link existed between hypoxia-induced alterations in contractile function and Bnip3 protein expression in vivo, we exposed neonatal WT and Bnip3^−/−^ mice to hypoxia [31]. Using transthoracic echocardiography, we observed that hypoxia induced significant contractile dysfunction in WT mice, including reductions in ejection fraction (EF), and alterations in left ventricular filling (E’/A’) (Fig. 7A–C), which remained unaffected in Bnip3^−/−^ mice (Fig. 7A–C). These results phenocopy what we observed using misoprostol drug treatments. In addition, we investigated the subcellular distribution of HMGB1 in neonatal hearts, and observed a decrease in nuclear localization in hypoxic WT animals, but was retained in the nucleus of hypoxia-exposed Bnip3^−/−^ mice (Fig. 7D).

## Discussion

Systemic hypoxia results in cardiac contractile impairment, resistance to inotropic therapy, end-organ perfusion defects, and contributes to the majority of neonatal deaths within the first week of life [50–56]. In this study we provide evidence that hypoxia-induced necroinflammation and neonatal cardiac dysfunction occur in a Bnip3-dependent manner. In addition, Bnip3 directly alters mitochondrial calcium homeostasis, resulting in MPT, necrosis and the release of the pro-inflammatory HMGB1 in the neonatal heart. Furthermore, we demonstrate that Bnip3 function can be pharmacologically modulated by misoprostol (see Fig. 7E for overview).

These results unify previous reports demonstrating the deleterious role of Bnip3 at multiple subcellular locations. At the ER/SR, we demonstrate that Bnip3 alters calcium homeostasis, consistent with previous studies where elevated mitochondrial calcium drives a loss of membrane potential, ROS production, MPT, and caspase-independent necrosis [8, 12, 57]. However, Bnip3 is a dual-regulator of cell death, where it also inserts through the outer mitochondrial membrane and uses its TM domain to interact with Opa1 [10, 58, 59]. While Opa1 is associated with maintaining cristae structure and mitochondrial fusion, genetic deletion of Opa1 results in ETC dysfunction, mitochondrial fragmentation and cell death [9, 60, 61]. Previous work demonstrates that cardiomyocyte-specific overexpression of Bnip3 results in complex-1 and -4 degradation, suppressing respiratory activity, while enhancing mitochondrial fragmentation [11]. ETC dysfunction is further tied to mitochondrial ROS production, which synergizes with MPT [62, 63]. Collectively, this suggests that Bnip3 functions to depress energy production and promote necrotic cell death in the heart through convergent pathways.

We also demonstrate that these pathways can be pharmacologically modulated through misoprostol-induced Bnip3 phosphorylation. While a previous study demonstrated that Bnip3 phosphorylation inhibits its interactions with Opa1, we provide mechanistic evidence both in vivo and in cardiomyocytes that misoprostol activates PKA, resulting in an inhibitory phosphorylation of Bnip3’s TM domain at Thr-181 [9]. Furthermore, we propose a mechanism by which 14-3-3β translocates Bnip3 to the cytosol, which likely prevents interactions with factors at ER and mitochondrial, including Bcl-2 and Opa1, respectively [64–68].

The results presented in this study strongly implicate Bnip3 as a regulator of mitochondrial calcium homeostasis and a necroinflammatory phenotype in the hypoxic neonatal heart. Additionally, the data in this preclinical study builds on the accumulating evidence that misoprostol directly regulates Bnip3 function, with potential meaningful implications for neonatal and adult hypoxia-induced cardiac pathologies, and stem cell-based cardiac therapies where promoting cardiomyocyte survival would be of benefit.

## Supplementary information

Supplemental

Confirmation of author list

Author checklist

## Data Availability

Authors can confirm that all relevant data are included in the article and/or its supplementary information files.
