# Supplementary material for: Misoprostol treatment prevents hypoxia-induced cardiac dysfunction through a 14-3-3 and PKA regulatory motif on Bnip3
Source: Cell Death Dis. 2021 Nov 26;12(12):1105. doi: 10.1038/s41419-021-04402-3 (PMC8617186; doi:10.1038/s41419-021-04402-3)
Supplement: Supplementary file 1 — Supplemental [file 41419_2021_4402_MOESM1_ESM.pdf]

Supplement 1. Complete list of relative Ct values for mitochondrial energy metabolism qRT-PCR array in PND10 heart samples (n=3 animals/condition), identified by mitochondrial complex.

Complex	Target	Array ID	NMX	HPX	HPX + MISO
Complex 1	Ndufa10	Mm00600325_m1	1.000000	0.197397	0.225888
	Ndufs4	Mm00656176_m1	1.000000	0.251290	0.626054
	Ndufa6	Mm01303455_g1	1.000000	0.446720	0.560977
	Ndufab1	Mm01137654_g1	1.000000	0.456153	0.530460
	Ndufa11	Mm01236867_g1	1.000000	0.462330	0.876922
	Ndufb3	Mm00835179_g1	1.000000	0.477876	5.086023
	Lhpp	Mm01269918_m1	1.000000	0.867248	2.086091
	Ndufb7	Mm00788005_s1	1.000000	0.883464	3.577282
	Ndufb9	Mm00612543_m1	1.000000	0.963204	4.114191
	Ndufs7	Mm01144210_m1	1.000000	0.993365	1.360459
	Ndufa5	Mm01165335_m1	1.000000	1.053928	2.232583
	Ndufa3	Mm01329704_g1	1.000000	1.119881	2.969799
	Ndufs3	Mm01329746_g1	1.000000	1.270824	0.955209
	Ndufs6	Mm02529639_u1	1.000000	1.272835	1.198779
	Ndufs5	Mm02600127_g1	1.000000	1.384180	3.076444
	Ndufv3	Mm01345700_m1	1.000000	1.870935	0.721520
	Ndufc2	Mm04213119_s1	1.000000	2.068060	1.442927
	Ndufs2	Mm00467603_g1	1.000000	2.454698	0.991675
	Ndufs1	Mm00523640_m1	1.000000	3.377395	2.718098
	Ndufb5	Mm00452592_m1	1.000000	5.968531	2.784598
Complex 2	Sdhd	Mm00546511_m1	1.000000	0.652135	0.805184
Complex 3	Cyc1	Mm00470540_m1	1.000000	1.051789	1.276995
	Uqcrq	Mm00772880_m1	1.000000	1.118767	0.921522
	Uqcr11	Mm00824470_g1	1.000000	1.519776	0.988861
	Uqcrc1	Mm00445911_m1	1.000000	2.289718	0.946879
	Uqcrh	Mm00835199_g1	1.000000	8.377721	0.874225
Complex 4	Cox11	Mm01615963_g1	1.000000	0.078951	0.971551
	Cox4i1	Mm01250094_m1	1.000000	0.676532	0.852358
	Cox5a	Mm00432638_m1	1.000000	0.860288	0.942858
	Cox7b	Mm00835076_g1	1.000000	1.087345	0.839040
	Cox7a2	Mm00438299_m1	1.000000	1.423320	2.855101
	Cox8c	Mm01325374_m1	1.000000	3.089582	0.000000
ATP Synthase	Atp5g3	Mm01334541_g1	1.000000	0.078880	0.130007
	Atp5d	Mm00502864_m1	1.000000	0.677803	4.668294
	Atp5a1	Mm00431960_m1	1.000000	4.229016	0.646722

Supplement 2. Complete list of relative Ct values for cell death pathway finder qRT-PCR array in PND10 heart samples (n=3 animals/condition), identified by cell death pathway.

Pathway	Target	Array ID	NMX	HPX	HPX + MISO
Apoptosis	Mcl1	Mm00725832_s1	1.00000	0.09085	0.23385
	Rab25	Mm00444175_m1	1.00000	0.26157	0.34519
	Cflar	Mm01255578_m1	1.00000	0.39314	0.71528
	Birc2	Mm00431811_m1	1.00000	0.81076	4.67332
	Bcl2	Mm00477631_m1	1.00000	2.16225	0.43035
	Xiap	Mm01311594_mH	1.00000	5.13869	5.39754
	Ywhaz	Mm01158417_g1	1.00000	8.17255	1.46103
	Bcl2l11	Mm00437796_m1	1.00000	0.33395	1.44004
	Casp1	Mm00438023_m1	1.00000	0.34537	1.73104
	Apaf1	Mm01223702_m1	1.00000	0.67758	1.14831
	Cd40	Mm00441891_m1	1.00000	2.10615	1.11862
	Casp2	Mm00432314_m1	1.00000	2.52140	5.98441
	Fas	Mm01204974_m1	1.00000	3.69722	4.33228
	Traf2	Mm00801978_m1	1.00000	3.90931	1.41652
	Dffa	Mm00438410_m1	1.00000	6.43212	0.40410
Autophagy	Esr1	Mm00433149_m1	1.00000	0.08536	0.12262
	Gaa	Mm00484581_m1	1.00000	0.17815	0.36807
	Akt1	Mm01331626_m1	1.00000	0.21520	0.26158
	Casp3	Mm01195085_m1	1.00000	0.23869	1.15156
	Pik3c3	Mm00619489_m1	1.00000	0.34942	0.36887
	Atg5	Mm00504340_m1	1.00000	0.53311	5.37482
	Irgm1	Mm00492596_m1	1.00000	0.59373	0.37320
	Map1lc3a	Mm00458725_g1	1.00000	0.66540	7.28435
	Atg7	Mm00512209_m1	1.00000	3.30256	0.76212
	Becn1	Mm01265461_m1	1.00000	3.62075	5.66199
	Atp6v1g2	Mm01159330_g1	1.00000	4.97828	6.60147
	Snca	Mm01188700_m1	1.00000	5.44339	5.97623
	Atg16l1	Mm00513085_m1	1.00000	5.48796	1.04288
	Mapk8	Mm00489514_m1	1.00000	8.34948	9.06761
Necrosis	Bmf	Mm00506773_m1	1.00000	0.16720	4.62077
	Cyld	Mm00557599_m1	1.00000	0.51276	8.72694
	Kcnip1	Mm01189526_m1	1.00000	0.19440	0.67120
	Dpysl4	Mm00496436_m1	1.00000	0.23837	2.38527
	Spata2	Mm00468039_g1	1.00000	0.31416	1.10926
	Ccdc103	Mm01248720_m1	1.00000	0.42559	1.86050
	Parp2	Mm00456462_m1	1.00000	0.55469	2.04393
	Parp1	Mm01321084_m1	1.00000	0.59457	0.56596
	Tmem57	Mm00550603_m1	1.00000	0.65448	1.23867
	Dennd4a	Mm00768872_m1	1.00000	5.50594	1.00824
Inflammation	Il12b	Mm01288992_m1	1.00000	0.27653	0.01620
	Edn1	Mm00438656_m1	1.00000	0.45629	0.40834
	Il1b	Mm00434228_m1	1.00000	0.49674	0.54534
	Il15	Mm00434210_m1	1.00000	2.05814	4.37305
	Cd4	Mm00442754_m1	1.00000	4.82734	2.44139
	Il12a	Mm00434165_m1	1.00000	10.19767	0.00000
	Il17a	Mm00439619_m1	1.00000	10.33179	0.00000

Supplemental 3.

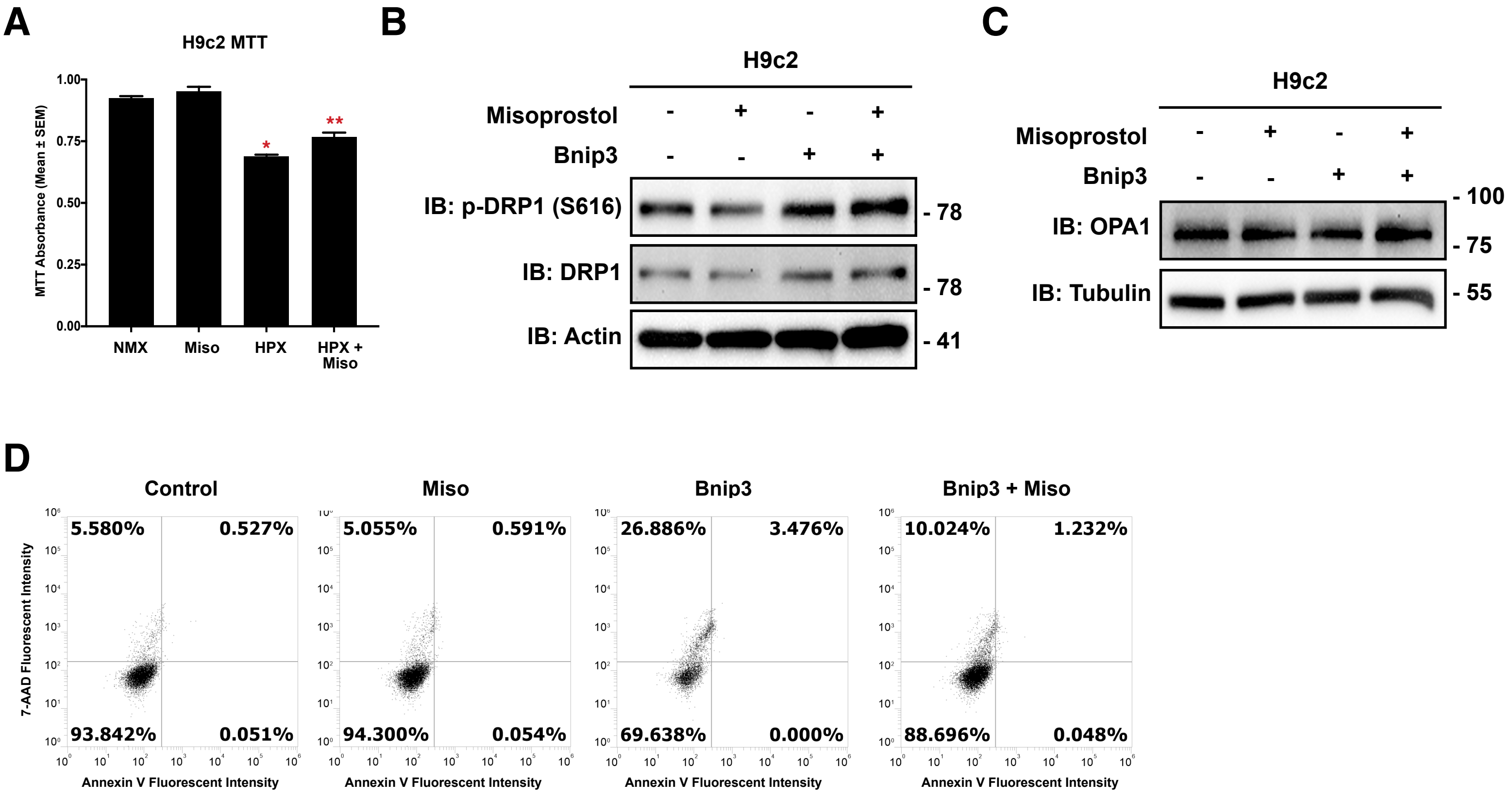

Supplement 3. Misoprostol prevents hypoxia- and Bnip3-induced cell death, but does not alter the expression of DRP1 or OPA1. (**A**) Quantification of H9c2 cells treated with 10 μM misoprostol (Miso) with or without exposure to 1% O_2 (HPX) for 24 hours. Cells were incubated with 3-(4,5-dimethylthiazol-2-yl)-2,5-diphenyltetrazolium bromide (MTT Reagent), and absorbance was assessed by plate reader. (**B**) Immunoblot for DRP1 expression in H9c2 cells transfected with pcDNA3 (control) or Myc-Bnip3 and treated with 10 μM misoprostol (Miso) or PBS control for 16 hours. **(C)** Immunoblot for OPA1 expression in H9c2 cells transfected with pcDNA3 (control) or Myc-Bnip3 and treated with 10 μM misoprostol (Miso) or PBS control for 16 hours. **(D)** Dot plots of 20,000 H9c2 cells transfected with pcDNA3 (control) or Myc-Bnip3 and treated with 10 μM misoprostol (Miso) or PBS control for 16 hours. Cells were stained with 7-AAD to indicate necrosis and Annexin V to indicate apoptosis. Cells were then analyzed by flow cytometry. All data are represented as mean ± S.E.M. **P*<0.05 compared with control, while ***P*<0.05 compared with hypoxia treatment, determined by 1-way ANOVA.

Supplemental 4.

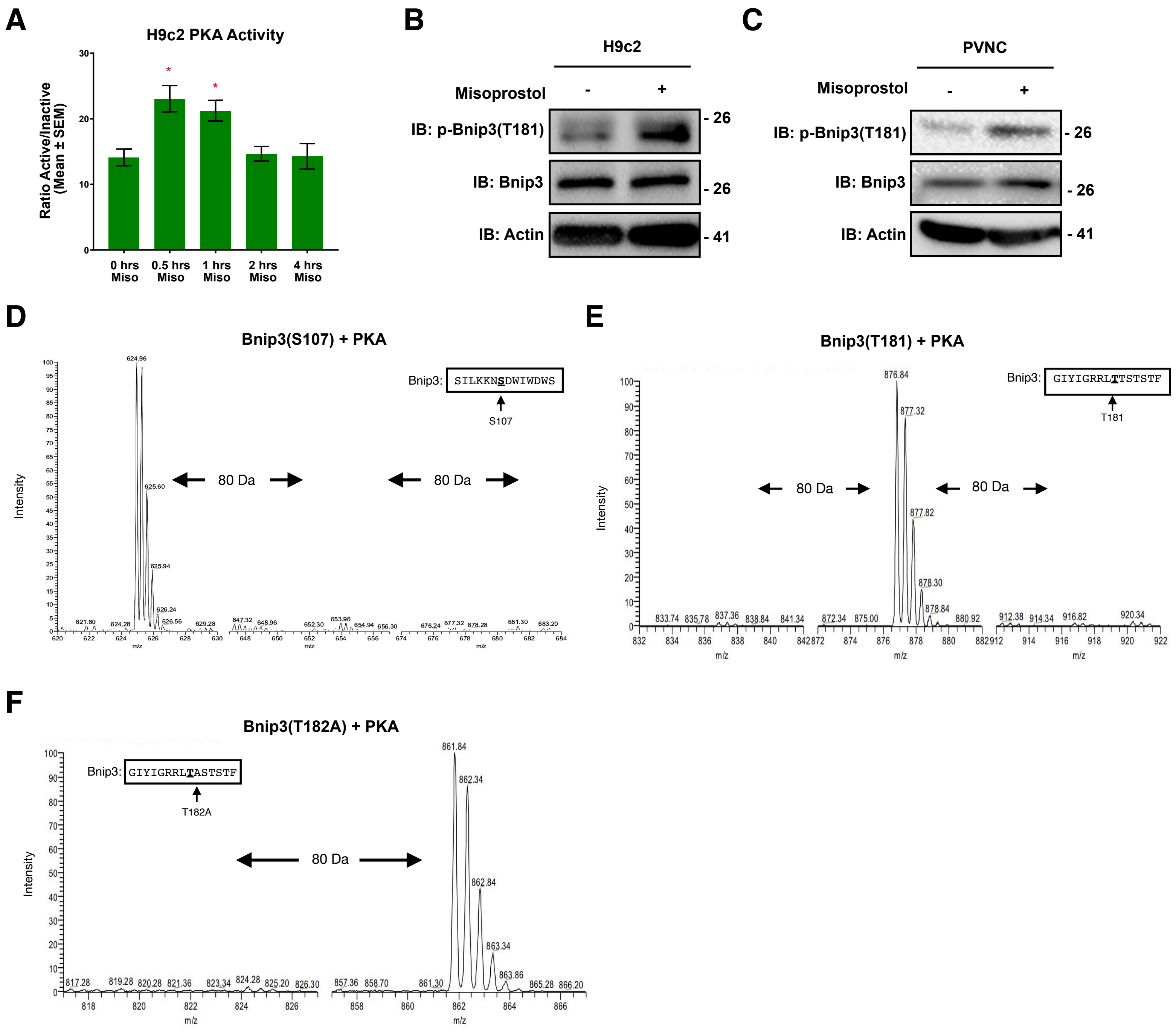

Supplement 4. Bnip3 phosphorylation by PKA. (A) Quantification of H9c2 cells transfected with pPHT-PKA and treated with 10 µM misoprostol for 0.5-h, 1-h, 2-h and 4-h. Cells were imaged by standard fluorescence microscopy and the ratio of green (active) to red (inactive) fluorescent signal was measured, normalized to cell area, and quantified in 10 random fields. **(B)** Immunoblot for phospho-Bnip3(T181) in protein extracts from H9c2 cells treated with 10 µM misoprostol or PBS control for 16 hours. **(C)** Immunoblot for phospho-Bnip3(T181) in protein extracts from PVNC's treated with 10 µM misoprostol or PBS control for 30 minutes. **(D)** SIM scan of the peptide spanning the predicted S107 PKA site of Bnip3. The unphosphorylated peptide has a 625 m/z (z=2+), which is not changed in the presence of PKA. **(E)** SIM scan of the wild-type peptide spanning the T181 PKA site of Bnip3. The unphosphorylated peptide has a 837 m/z (z=2+) which is shifted by phosphorylation showing an increased m/z of 20 that corresponds to a single PO3 (M = 80.00 Da) and not a double phosphorylation (M=160.00 Da). **(F)** SIM scan of a mutated peptide where the PKA site at Threonine-182 is replaced with Alanine. The unphosphorylated peptide has a 837 m/z (z=2+), while putative phosphorylation shows an increased m/z of 20 that corresponds to PO3 (M = 80.00 Da), demonstrating no effect of T182A on PKA's ability to phosphorylate Bnip3. All data are represented as mean ± S.E.M. *P<0.05 compared with control determined by 1-way ANOVA.

Supplemental 5.

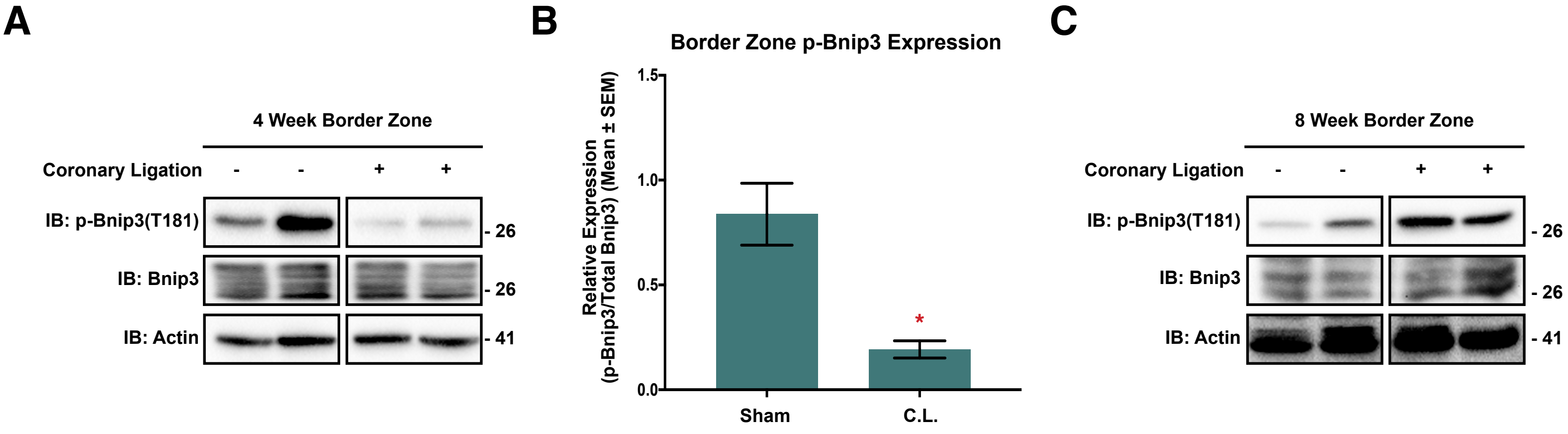

Supplement 5. Phosphorylation of Bnip3 at Thr-181 is altered in adult hypoxia-induced pathologies. (A) Representative immunoblot of heart protein extracts from Sprague Dawley rats subjected to left coronary artery ligation, or sham operation as a control. Following 4-weeks of recovery, the viable infarct border-zone was harvested from the left ventricle. Extracts were immunoblotted for phospho-Bnip3 expression. **(B)** Densitometry analysis for extracts in (A), representing an N of 4 animals per condition. **(C)** Representative immunoblot of heart protein extracts from Sprague Dawley rats treated as in (A) and viable infarct border-zone was harvested 8-weeks of recovery. Extracts were immunoblotted for phospho-Bnip3 expression. All data are represented as mean ± S.E.M. *P<0.05 compared with control determined by Students T-Test.

Supplemental 6.

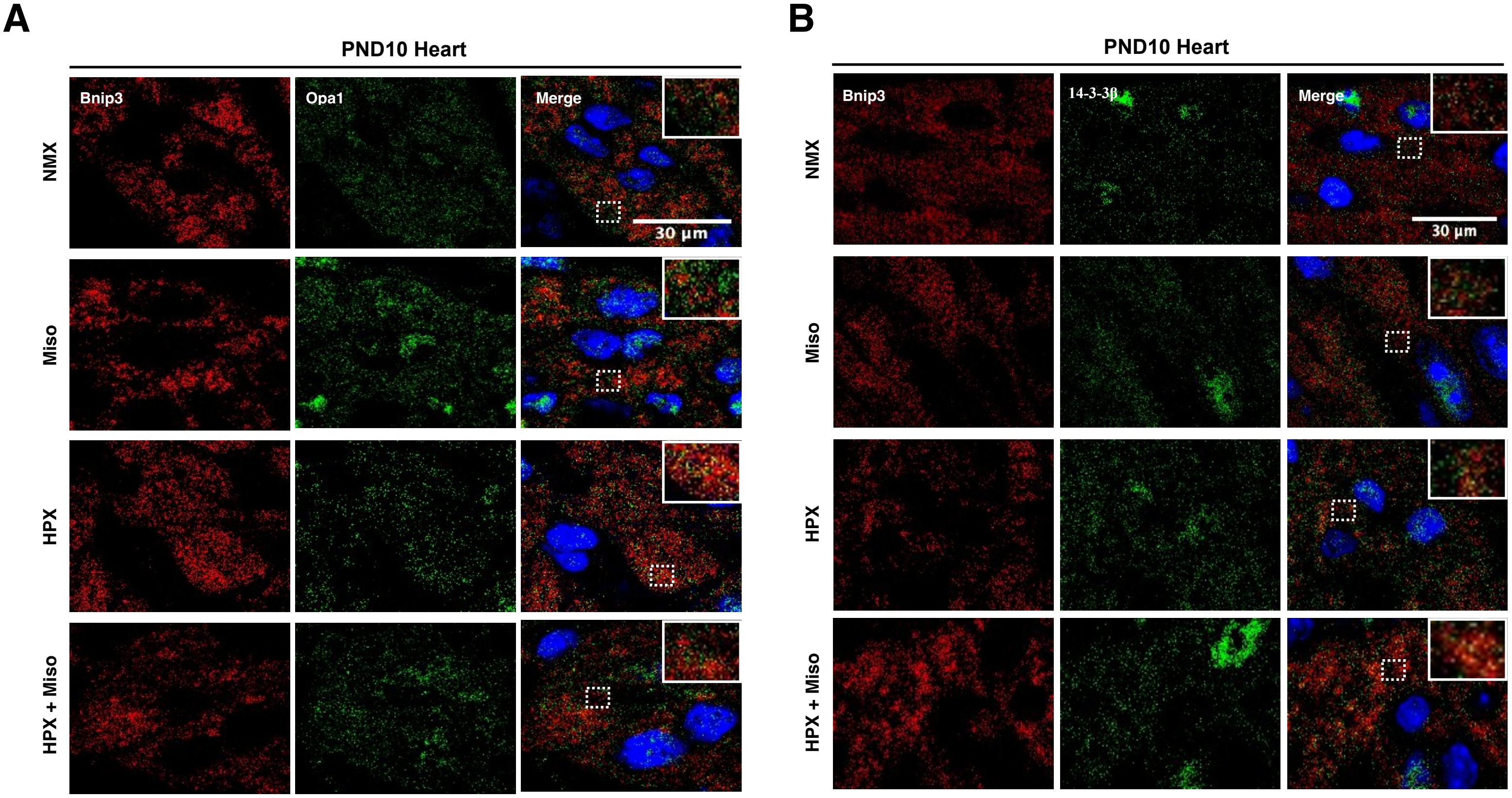

Supplement 6. Misoprostol alters Bnip3's interactions with Opa1 and 14-3-3β. (A) PND10 hearts from mice exposed to hypoxia (10% O_2) ± 10 µg/kg misoprostol from PND3-10, stained with DAPI (Blue) and probed for Bnip3 (Red), and Opa1 (green). Hearts were imaged via confocal microscopy. **(B)** PND10 hearts from mice treated as in (A) and stained with DAPI (Blue) and probed for Bnip3 (Red), and 14-3-3β (green). Hearts were imaged via confocal microscopy.

Supplemental 7.

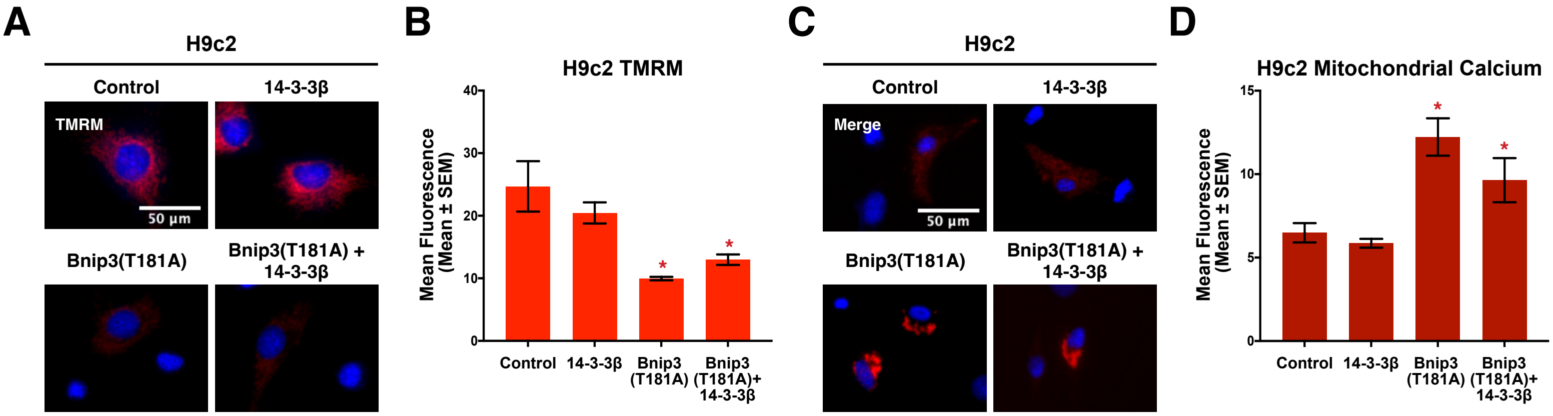

Supplement 7. 14-3-3β fails to prevent Bnip3-induced mitochondrial depolarization and calcium accumulation when the Thr-181 phosphorylation site is missing in H9c2 cells. (A) H9c2 cells transfected with pcDNA3 (control) or Myc-Bnip3(T181A) with and without HA-14-3-3β. Cells were stained with TMRM (red) and hoechst (blue) and imaged by standard fluorescence microscopy. **(B)** Quantification of cells in (A), where red fluorescent signal was normalized to cell area and quantified in 10 random fields. **(C)** H9c2 cells transfected with pcDNA3 (control) or Myc-Bnip3(T181A) with and without HA-14-3-3β. Mito-CAR-GECO (red) was included in all conditions to assess mitochondrial calcium content. Cells were stained with hoechst (blue) and imaged by standard fluorescence microscopy. **(D)** Quantification of cells in (C), where red fluorescent signal was normalized to cell area and quantified in 10 random fields. All data are represented as mean ± S.E.M. *$P<0.05$ compared with control, while **$P<0.05$ compared with hypoxia treatment, determined by 1-way ANOVA.
