# Supplementary material for: Misoprostol treatment prevents hypoxia-induced cardiac dysfunction through a 14-3-3 and PKA regulatory motif on Bnip3
Source: Cell Death Dis. 2021 Nov 26;12(12):1105. doi: 10.1038/s41419-021-04402-3 (PMC8617186; doi:10.1038/s41419-021-04402-3)
Supplement: Supplementary file 2 — Confirmation of author list [file 41419_2021_4402_MOESM2_ESM.pdf]

From: Rampitsch, Christof chris.rampitsch@AGR.GC.CA
Subject: RE: CDDIS-21-1957RRR Initial Quality Check
Date: November 4, 2021 at 12:27 PM
To: Joe Gordon Joseph.Gordon@umanitoba.ca

Confirm additional authors.

Chris.
Dr. Christof Rampitsch
Research Scientist/ Chargé de Recherche
Morden Research and Development Centre / Centre de recherche et de développement de Morden
Agriculture and Agrifood Canada/Agriculture et agroalimentaire Canada
101 Route 100
Morden MB
Canada R6M 1Y5

Tel./Tél. 204 822-7602
Cell. 204 229-9034

From: Joe Gordon <Joseph.Gordon@umanitoba.ca>
Sent: Thursday, November 4, 2021 10:27 AM
To: Matthew Martens <marten22@myumanitoba.ca>; nivedi87@gmail.com; Nguyen Lucas <nguyenlucas90@gmail.com>; Donald Chapman <DChapman@chrim.ca>; Elizabeth Henson <Elizabeth.Henson@umanitoba.ca>; Bo Xiang <BXiang@chrim.ca>; landonfalk <landonfalk@gmail.com>; arielysm@bcm.edu; Sunil Rattan <SRattan@sbrc.ca>; Jared Field <umfiel26@myumanitoba.ca>; Philip Kawalec <kawalecp@myumanitoba.ca>; Spencer Gibson <Spencer.Gibson@umanitoba.ca>; Richard Keijzer <RKeijzer@chrim.ca>; Ayesha Saleem <Ayesha.Saleem@umanitoba.ca>; Grant Hatch <GHatch@chrim.ca>; Christine Doucette <CDoucette@chrim.ca>; Jason Karch <Jason.karch@bcm.edu>; Vern Dolinsky <Vernon.Dolinsky@umanitoba.ca>; Ian Dixon <idixon@sbrc.ca>; Adrian West <Adrian.West@umanitoba.ca>; Rampitsch, Christof <chris.rampitsch@AGR.GC.CA>
Subject: Fwd: CDDIS-21-1957RRR Initial Quality Check
Importance: High

Hello everyone,

During the review process the author list for Matt's paper changed (see below). Most notably, Jared Field and Philip Kawalec were added for their assistance with the flow cytometry experiments.

The paper has been accepted, but the journal (Cell Death and Disease) needs confirmation that all authors agree to this authorship change.

Please reply as soon as you can with a simple "Confirm" and I will compile and upload.

Thank you for all your support.

Joe.

Matthew D. Martens[1,8], Nivedita Seshadri[2,8], Lucas Nguyen[8], Donald Chapman[8], Elizabeth S. Henson[3,10], Bo Xiang[4,8], Landon Falk[2,9], Arielys Mendoza[12], Sunil Rattan[4,11], Jared T. Field[1,8], Philip Kawalec[1,8], Spencer B. Gibson[3,10], Richard Keijzer[5,9], Ayesha Saleem[7,8], Grant M. Hatch[4,8], Christine A. Doucette[2,8], Jason M. Karch[12], Vernon W. Dolinsky[4,8], Ian M. Dixon[2,11], Adrian R. West[2,9], Christof Rampitsch[13], and Joseph W. Gordon[1,6,8,*]

Begin forwarded message:

From: cddisease@springernature.com
Subject: CDDIS-21-1957RRR Initial Quality Check
Date: November 4, 2021 at 9:36:18 AM ADT
To: joseph.gordon@umanitoba.ca
Reply-To: cddisease@springernature.com

Caution: This message was sent from outside the University of Manitoba.

Dear Dr Gordon,

In checking in your manuscript submitted to Cell Death & Disease it has come to our attention that the following must be addressed before we can proceed.

1. Please make sure you include a data availability statement in your Article file under its own subheading.

2. Please make sure all tables and figures are cited in the main text, in numerical order

3. It has come to our attention that your most recent author list differs from the one in your original submission.

Please request agreement from all authors including additions and deletions, these can be collected in the following way:

Email your co-authors with the change, and ask them to reply to your email confirming that they agree to these changes. Once you have collected these replies, please combine all of the co-authors' email responses in one document and upload this file to your submission.

Your paper is now available for you to edit, you may access via the following link:

https://mts-cddis.nature.com/cgi-bin/main.plex?el=A2CA6UZA6D2dOU5F1A9ftdqTfhMrqgyuhG60SwflYg7QZ

(Press/Click on the above link to be automatically sent to the web page.)

Please make the correction(s) as specified above and resubmit your paper following the same steps as before.

If you have any questions please feel free to contact us.

Sincerely,

Editorial Office
Cell Death & Disease
cddisease@springernature.com

Our flexible approach during the COVID-19 pandemic
If you need more time at any stage of the peer-review process, please do let us know. While our systems will continue to remind you of the original timelines, we aim to be as flexible as possible during the current pandemic.

This email has been sent through the NPG Manuscript Tracking System NY-610A-NPG&MTS

Confidentiality Statement:

This e-mail is confidential and subject to copyright. Any unauthorised use or disclosure of its contents is prohibited. If you have received this email in error please notify our Manuscript Tracking System Helpdesk team at http://platformsupport.nature.com .
Details of the confidentiality and pre-publicity policy may be found here http://www.nature.com/authors/policies/confidentiality.html
Privacy Policy | Update Profile

Caution: This message was sent from outside the University of Manitoba.

From: **Adrian West** Adrian.West@umanitoba.ca
Subject: Re: CDDIS-21-1957RRR Initial Quality Check
Date: November 4, 2021 at 2:50 PM
To: Joe Gordon Joseph.Gordon@umanitoba.ca

AW

Confirm!

From: Joe Gordon <Joseph.Gordon@umanitoba.ca>
Sent: Thursday, November 4, 2021, 10:26 a.m.
To: Matthew Martens; nivedi87@gmail.com; Nguyen Lucas; Donald Chapman; Elizabeth Henson; Bo Xiang; landonfalk; arielysm@bcm.edu; Sunil Rattan; Jared Field; Philip Kawalec; Spencer Gibson; Richard Keijzer; Ayesha Saleem; Grant Hatch; Christine Doucette; Jason Karch; Vern Dolinsky; Ian Dixon; Adrian West; Christof Rampitsch
Subject: Fwd: CDDIS-21-1957RRR Initial Quality Check

Hello everyone,

During the review process the author list for Matt's paper changed (see below). Most notably, Jared Field and Philip Kawalec were added for their assistance with the flow cytometry experiments.

The paper has been accepted, but the journal (Cell Death and Disease) needs confirmation that all authors agree to this authorship change.

Please reply as soon as you can with a simple "Confirm" and I will compile and upload.

Thank you for all your support.

Joe.

Matthew D. Martens[1,8], Nivedita Seshadri[2,8], Lucas Nguyen[8], Donald Chapman[8], Elizabeth S. Henson[3,10], Bo Xiang[4,8], Landon Falk[2,9], Arielys Mendoza[12], Sunil Rattan[4,11], Jared T. Field[1,8], Philip Kawalec[1,8], Spencer B. Gibson[3,10], Richard Keijzer[5,9], Ayesha Saleem[7,8], Grant M. Hatch[4,8], Christine A. Doucette[2,8], Jason M. Karch[12], Vernon W. Dolinsky[4,8], Ian M. Dixon[2,11], Adrian R. West[2,9], Christof Rampitsch[13], and Joseph W. Gordon[1,6,8,*]

> Begin forwarded message:
>
> **From:** cddisease@springernature.com
> **Subject:** **CDDIS-21-1957RRR Initial Quality Check**
> **Date:** November 4, 2021 at 9:36:18 AM ADT
> **To:** joseph.gordon@umanitoba.ca
> **Reply-To:** cddisease@springernature.com
>
> **Caution:** This message was sent from outside the University of Manitoba.
>
> Dear Dr Gordon,
>
> In checking in your manuscript submitted to Cell Death & Disease it has come to our attention that the following must be addressed before we can proceed.
>
> 1. Please make sure you include a data availability statement in your Article file under its own subheading.
>
> 2. Please make sure all tables and figures are cited in the main text, in numerical order
>
> 3. It has come to our attention that your most recent author list differs from the one in your original submission.
>
> Please request agreement from all authors including additions and deletions, these can be collected in the following way:
>
> Email your co-authors with the change, and ask them to reply to your email confirming that they agree to these changes. Once you have collected these replies, please combine all of the co-authors' email responses in one document and upload this file to your submission.
>
> Your paper is now available for you to edit, you may access via the following link:
>
> https://mts-cddis.nature.com/cgi-bin/main.plex?el=A2CA6UZA6D2dOU5F1A9ftdqTfhMrqgyuhG60SwflYg7QZ

(Press/Click on the above link to be automatically sent to the web page.)

Please make the correction(s) as specified above and resubmit your paper following the same steps as before.

If you have any questions please feel free to contact us.

Sincerely,

Editorial Office
Cell Death & Disease
cddisease@springernature.com

Our flexible approach during the COVID-19 pandemic
If you need more time at any stage of the peer-review process, please do let us know. While our systems will continue to remind you of the original timelines, we aim to be as flexible as possible during the current pandemic.

This email has been sent through the NPG Manuscript Tracking System NY-610A-NPG&MTS

Confidentiality Statement:

This e-mail is confidential and subject to copyright. Any unauthorised use or disclosure of its contents is prohibited. If you have received this email in error please notify our Manuscript Tracking System Helpdesk team at http://platformsupport.nature.com .

Details of the confidentiality and pre-publicity policy may be found here http://www.nature.com/authors/policies/confidentiality.html

Privacy Policy | Update Profile

From: Mendoza, Arielys Melissa Arielys.Mendoza@bcm.edu
Subject: Re: CDDIS-21-1957RRR Initial Quality Check
Date: November 4, 2021 at 12:33 PM
To: Joe Gordon Joseph.Gordon@umanitoba.ca

AM

Caution: This message was sent from outside the University of Manitoba.

confirm

Arielys Mendoza
PhD Candidate
Dr. Jason Karch Lab
Molecular Physiology and Biophysics
Baylor College of Medicine

From: Joe Gordon
Sent: Thursday, November 4, 2021 10:26 AM
To: Matthew Martens; nivedi87@gmail.com; Nguyen Lucas; Donald Chapman; Elizabeth Henson; Bo Xiang; landonfalk; Mendoza, Arielys Melissa; Sunil Rattan; Jared Field; Philip Kawalec; Spencer Gibson; Richard Keijzer; Ayesha Saleem; Grant Hatch; Christine Doucette; Karch, Jason M.; Vern Dolinsky; Ian Dixon; Adrian West
Subject: Fwd: CDDIS-21-1957RRR Initial Quality Check

*****CAUTION:*** This email is not from a BCM Source. Only click links or open attachments you know are safe.**

Hello everyone,

During the review process the author list for Matt's paper changed (see below). Most notably, Jared Field and Philip Kawalec were added for their assistance with the flow cytometry experiments.

The paper has been accepted, but the journal (Cell Death and Disease) needs confirmation that all authors agree to this authorship change.

Please reply as soon as you can with a simple "Confirm" and I will compile and upload.

Thank you for all your support.

Joe.

Matthew D. Martens[1,8], Nivedita Seshadri[2,8], Lucas Nguyen[8], Donald Chapman[8], Elizabeth S. Henson[3,10], Bo Xiang[4,8], Landon Falk[2,9], Arielys Mendoza[12], Sunil Rattan[4,11], Jared T. Field[1,8], Philip Kawalec[1,8], Spencer B. Gibson[3,10], Richard Keijzer[5,9], Ayesha Saleem[7,8], Grant M. Hatch[4,8], Christine A. Doucette[2,8], Jason M. Karch[12], Vernon W. Dolinsky[4,8], Ian M. Dixon[2,11], Adrian R. West[2,9], Christof Rampitsch[13], and Joseph W. Gordon[1,6,8,*]

> Begin forwarded message:
>
> **From:** cddisease@springernature.com
> **Subject:** **CDDIS-21-1957RRR Initial Quality Check**
> **Date:** November 4, 2021 at 9:36:18 AM ADT
> **To:** joseph.gordon@umanitoba.ca
> **Reply-To:** cddisease@springernature.com
>
> **Caution:** This message was sent from outside the University of Manitoba.
>
> Dear Dr Gordon,

In checking in your manuscript submitted to Cell Death & Disease it has come to our attention that the following must be addressed before we can proceed.

1. Please make sure you include a data availability statement in your Article file under its own subheading.

2. Please make sure all tables and figures are cited in the main text, in numerical order

3. It has come to our attention that your most recent author list differs from the one in your original submission.

Please request agreement from all authors including additions and deletions, these can be collected in the following way:

Email your co-authors with the change, and ask them to reply to your email confirming that they agree to these changes. Once you have collected these replies, please combine all of the co-authors' email responses in one document and upload this file to your submission.

Your paper is now available for you to edit, you may access via the following link:

https://mts-cddis.nature.com/cgi-bin/main.plex?el=A2CA6UZA6D2dOU5F1A9ftdqTfhMrqgyuhG60SwflYg7QZ

(Press/Click on the above link to be automatically sent to the web page.)

Please make the correction(s) as specified above and resubmit your paper following the same steps as before.

If you have any questions please feel free to contact us.

Sincerely,

Editorial Office
Cell Death & Disease
cddisease@springernature.com

Our flexible approach during the COVID-19 pandemic
If you need more time at any stage of the peer-review process, please do let us know. While our systems will continue to remind you of the original timelines, we aim to be as flexible as possible during the current pandemic.

This email has been sent through the NPG Manuscript Tracking System NY-610A-NPG&MTS

Confidentiality Statement:

This e-mail is confidential and subject to copyright. Any unauthorised use or disclosure of its contents is prohibited. If you have received this email in error please notify our Manuscript Tracking System Helpdesk team at http://platformsupport.nature.com . Details of the confidentiality and pre-publicity policy may be found here http://www.nature.com/authors/policies/confidentiality.html
Privacy Policy | Update Profile

From: Ayesha Saleem Ayesha.Saleem@umanitoba.ca
Subject: Re: CDDIS-21-1957RRR Initial Quality Check
Date: November 4, 2021 at 12:51 PM
To: Joe Gordon Joseph.Gordon@umanitoba.ca

AS

confirm.

Ayesha Saleem, PhD
Assistant Professor
Faculty of Kinesiology and Recreation Management (FKRM)
120 Frank Kennedy Centre, University of Manitoba, Winnipeg, MB, R3T 2N2
204.474.7617 | ayesha.saleem@umanitoba.ca | https://umanitoba.ca/faculties/kinrec/about/saleem.html

Principal Investigator
Children's Hospital Research Institute of Manitoba (CHRIM)
600A - 715 McDermot Avenue, John Buhler Research Centre (JBRC), Winnipeg, MB R3E 3P4
204-789-3688 | asaleem@chrim.ca | https://www.chrim.ca/investigator/ayesha-saleem/

Lab: 631 JBRC | 204-789-3561 | https://umanitoba.ca/faculties/kinrec/research/evelab.html

---

From: Joe Gordon
Sent: Thursday, November 4, 2021 10:26 AM
To: Matthew Martens; nivedi87@gmail.com; Nguyen Lucas; Donald Chapman; Elizabeth Henson; Bo Xiang; landonfalk; arielysm@bcm.edu; Sunil Rattan; Jared Field; Philip Kawalec; Spencer Gibson; Richard Keijzer; Ayesha Saleem; Grant Hatch; Christine Doucette; Jason Karch; Vern Dolinsky; Ian Dixon; Adrian West
Subject: Fwd: CDDIS-21-1957RRR Initial Quality Check

Hello everyone,

During the review process the author list for Matt's paper changed (see below). Most notably, Jared Field and Philip Kawalec were added for their assistance with the flow cytometry experiments.

The paper has been accepted, but the journal (Cell Death and Disease) needs confirmation that all authors agree to this authorship change.

Please reply as soon as you can with a simple "Confirm" and I will compile and upload.

Thank you for all your support.

Joe.

Matthew D. Martens[1,8], Nivedita Seshadri[2,8], Lucas Nguyen[8], Donald Chapman[8], Elizabeth S. Henson[3,10], Bo Xiang[4,8], Landon Falk[2,9], Arielys Mendoza[12], Sunil Rattan[4,11], Jared T. Field[1,8], Philip Kawalec[1,8], Spencer B. Gibson[3,10], Richard Keijzer[5,9], Ayesha Saleem[7,8], Grant M. Hatch[4,8], Christine A. Doucette[2,8], Jason M. Karch[12], Vernon W. Dolinsky[4,8], Ian M. Dixon[2,11], Adrian R. West[2,9], Christof Rampitsch[13], and Joseph W. Gordon[1,6,8,*]

> Begin forwarded message:
>
> **From:** cddisease@springernature.com
> **Subject:** **CDDIS-21-1957RRR Initial Quality Check**
> **Date:** November 4, 2021 at 9:36:18 AM ADT
> **To:** joseph.gordon@umanitoba.ca
> **Reply-To:** cddisease@springernature.com
>
> **Caution:** This message was sent from outside the University of Manitoba.
>
> Dear Dr Gordon

Dear Dr Gordon,

In checking in your manuscript submitted to Cell Death & Disease it has come to our attention that the following must be addressed before we can proceed.

1. Please make sure you include a data availability statement in your Article file under its own subheading.

2. Please make sure all tables and figures are cited in the main text, in numerical order

3. It has come to our attention that your most recent author list differs from the one in your original submission.

Please request agreement from all authors including additions and deletions, these can be collected in the following way:

Email your co-authors with the change, and ask them to reply to your email confirming that they agree to these changes. Once you have collected these replies, please combine all of the co-authors' email responses in one document and upload this file to your submission.

Your paper is now available for you to edit, you may access via the following link:

https://mts-cddis.nature.com/cgi-bin/main.plex?el=A2CA6UZA6D2dOU5F1A9ftdqTfhMrqgyuhG60SwflYg7QZ

(Press/Click on the above link to be automatically sent to the web page.)

Please make the correction(s) as specified above and resubmit your paper following the same steps as before.

If you have any questions please feel free to contact us.

Sincerely,

Editorial Office
Cell Death & Disease
cddisease@springernature.com

Our flexible approach during the COVID-19 pandemic
If you need more time at any stage of the peer-review process, please do let us know. While our systems will continue to remind you of the original timelines, we aim to be as flexible as possible during the current pandemic.

This email has been sent through the NPG Manuscript Tracking System NY-610A-NPG&MTS

Confidentiality Statement:

This e-mail is confidential and subject to copyright. Any unauthorised use or disclosure of its contents is prohibited. If you have received this email in error please notify our Manuscript Tracking System Helpdesk team at http://platformsupport.nature.com . Details of the confidentiality and pre-publicity policy may be found here http://www.nature.com/authors/policies/confidentiality.html
Privacy Policy | Update Profile

From: **Christine Doucette** CDoucette@chrim.ca
Subject: Re: CDDIS-21-1957RRR Initial Quality Check
Date: November 4, 2021 at 12:34 PM
To: Joe Gordon Joseph.Gordon@umanitoba.ca

CD

Confirm.

> On Nov 4, 2021, at 8:26 AM, Joe Gordon <Joseph.Gordon@umanitoba.ca> wrote:
>
> Hello everyone,
>
> During the review process the author list for Matt's paper changed (see below). Most notably, Jared Field and Philip Kawalec were added for their assistance with the flow cytometry experiments.
>
> The paper has been accepted, but the journal (Cell Death and Disease) needs confirmation that all authors agree to this authorship change.
>
> Please reply as soon as you can with a simple "Confirm" and I will compile and upload.
>
> Thank you for all your support.
>
> Joe.
>
> Matthew D. Martens[1,8], Nivedita Seshadri[2,8], Lucas Nguyen[8], Donald Chapman[8], Elizabeth S. Henson[3,10], Bo Xiang[4,8], Landon Falk[2,9], Arielys Mendoza[12], Sunil Rattan[4,11], Jared T. Field[1,8], Philip Kawalec[1,8], Spencer B. Gibson[3,10], Richard Keijzer[5,9], Ayesha Saleem[7,8], Grant M. Hatch[4,8], Christine A. Doucette[2,8], Jason M. Karch[12], Vernon W. Dolinsky[4,8], Ian M. Dixon[2,11], Adrian R. West[2,9], Christof Rampitsch[13], and Joseph W. Gordon[1,6,8,*]
>
>> Begin forwarded message:
>>
>> **From:** cddisease@springernature.com
>> **Subject: CDDIS-21-1957RRR Initial Quality Check**
>> **Date:** November 4, 2021 at 9:36:18 AM ADT
>> **To:** joseph.gordon@umanitoba.ca
>> **Reply-To:** cddisease@springernature.com
>>
>> **Caution:** This message was sent from outside the University of Manitoba.
>>
>> Dear Dr Gordon,
>>
>> In checking in your manuscript submitted to Cell Death & Disease it has come to our attention that the following must be addressed before we can proceed.
>>
>> 1. Please make sure you include a data availability statement in your Article file under its own subheading.
>>
>> 2. Please make sure all tables and figures are cited in the main text, in numerical order
>>
>> 3. It has come to our attention that your most recent author list differs from the one in your original submission.
>>
>> Please request agreement from all authors including additions and deletions, these can be collected in the following way:
>>
>> Email your co-authors with the change, and ask them to reply to your email confirming that they agree to these changes. Once you have collected these replies, please combine all of the co-authors' email responses in one document and upload this file to your submission.
>>
>> Your paper is now available for you to edit, you may access via the following link:
>>
>> https://mts-cddis.nature.com/cgi-bin/main.plex?el=A2CA6UZA6D2dOU5F1A9ftdqTfhMrqgyuhG60SwflYg7QZ
>>
>> (Press/Click on the above link to be automatically sent to the web page.)
>>
>> Please make the correction(s) as specified above and resubmit your paper following the same steps as before.
>>
>> If you have any questions please feel free to contact us.

Sincerely,

Editorial Office
Cell Death & Disease
cddisease@springernature.com

Our flexible approach during the COVID-19 pandemic
If you need more time at any stage of the peer-review process, please do let us know. While our systems will continue to remind you of the original timelines, we aim to be as flexible as possible during the current pandemic.

This email has been sent through the NPG Manuscript Tracking System NY-610A-NPG&MTS

Confidentiality Statement:

This e-mail is confidential and subject to copyright. Any unauthorised use or disclosure of its contents is prohibited. If you have received this email in error please notify our Manuscript Tracking System Helpdesk team at http://platformsupport.nature.com .

Details of the confidentiality and pre-publicity policy may be found here http://www.nature.com/authors/policies/confidentiality.html

Privacy Policy | Update Profile

From: **Donald Chapman** dchapman@chrim.ca
Subject: Re: CDDIS-21-1957RRR Initial Quality Check
Date: November 4, 2021 at 1:07 PM
To: Joe Gordon Joseph.Gordon@umanitoba.ca

DC

Confirm,

Thank you,

Donald Chapman

From: Joe Gordon
Sent: Thursday, November 4, 2021 3:26 PM
To: Matthew Martens; nivedi87@gmail.com; Nguyen Lucas; Donald Chapman; Elizabeth Henson; Bo Xiang; landonfalk; arielysm@bcm.edu; Sunil Rattan; Jared Field; Philip Kawalec; Spencer Gibson; Richard Keijzer; Ayesha Saleem; Grant Hatch; Christine Doucette; Jason Karch; Vern Dolinsky; Ian Dixon; Adrian West
Subject: Fwd: CDDIS-21-1957RRR Initial Quality Check

Hello everyone,

During the review process the author list for Matt's paper changed (see below). Most notably, Jared Field and Philip Kawalec were added for their assistance with the flow cytometry experiments.

The paper has been accepted, but the journal (Cell Death and Disease) needs confirmation that all authors agree to this authorship change.

Please reply as soon as you can with a simple "Confirm" and I will compile and upload.

Thank you for all your support.

Joe.

Matthew D. Martens[1,8], Nivedita Seshadri[2,8], Lucas Nguyen[8], Donald Chapman[8], Elizabeth S. Henson[3,10], Bo Xiang[4,8], Landon Falk[2,9], Arielys Mendoza[12], Sunil Rattan[4,11], Jared T. Field[1,8], Philip Kawalec[1,8], Spencer B. Gibson[3,10], Richard Keijzer[5,9], Ayesha Saleem[7,8], Grant M. Hatch[4,8], Christine A. Doucette[2,8], Jason M. Karch[12], Vernon W. Dolinsky[4,8], Ian M. Dixon[2,11], Adrian R. West[2,9], Christof Rampitsch[13], and Joseph W. Gordon[1,6,8,*]

> Begin forwarded message:
>
> **From:** cddisease@springernature.com
> **Subject:** **CDDIS-21-1957RRR Initial Quality Check**
> **Date:** November 4, 2021 at 9:36:18 AM ADT
> **To:** joseph.gordon@umanitoba.ca
> **Reply-To:** cddisease@springernature.com
>
> **Caution:** This message was sent from outside the University of Manitoba.
>
> Dear Dr Gordon,
>
> In checking in your manuscript submitted to Cell Death & Disease it has come to our attention that the following must be addressed before we can proceed.
>
> 1. Please make sure you include a data availability statement in your Article file under its own subheading.

2. Please make sure all tables and figures are cited in the main text, in numerical order

3. It has come to our attention that your most recent author list differs from the one in your original submission.

Please request agreement from all authors including additions and deletions, these can be collected in the following way:

Email your co-authors with the change, and ask them to reply to your email confirming that they agree to these changes. Once you have collected these replies, please combine all of the co-authors' email responses in one document and upload this file to your submission.

Your paper is now available for you to edit, you may access via the following link:

https://mts-cddis.nature.com/cgi-bin/main.plex?el=A2CA6UZA6D2dOU5F1A9ftdqTfhMrqgyuhG60SwflYg7QZ

(Press/Click on the above link to be automatically sent to the web page.)

Please make the correction(s) as specified above and resubmit your paper following the same steps as before.

If you have any questions please feel free to contact us.

Sincerely,

Editorial Office
Cell Death & Disease
cddisease@springernature.com

Our flexible approach during the COVID-19 pandemic
If you need more time at any stage of the peer-review process, please do let us know. While our systems will continue to remind you of the original timelines, we aim to be as flexible as possible during the current pandemic.

This email has been sent through the NPG Manuscript Tracking System NY-610A-NPG&MTS

Confidentiality Statement:

This e-mail is confidential and subject to copyright. Any unauthorised use or disclosure of its contents is prohibited. If you have received this email in error please notify our Manuscript Tracking System Helpdesk team at http://platformsupport.nature.com . Details of the confidentiality and pre-publicity policy may be found here http://www.nature.com/authors/policies/confidentiality.html
Privacy Policy | Update Profile

From: **Grant Hatch** GHatch@chrim.ca
Subject: Re: CDDIS-21-1957RRR Initial Quality Check
Date: November 4, 2021 at 12:45 PM
To: Joe Gordon Joseph.Gordon@umanitoba.ca

Dear Joe
I approve the authorship change.
Sincerely
Dr. Grant M Hatch

Sent from my iPhone

> On Nov 4, 2021, at 10:26 AM, Joe Gordon <Joseph.Gordon@umanitoba.ca> wrote:
>
> Hello everyone,
>
> During the review process the author list for Matt's paper changed (see below). Most notably, Jared Field and Philip Kawalec were added for their assistance with the flow cytometry experiments.
>
> The paper has been accepted, but the journal (Cell Death and Disease) needs confirmation that all authors agree to this authorship change.
>
> Please reply as soon as you can with a simple "Confirm" and I will compile and upload.
>
> Thank you for all your support.
>
> Joe.
>
> Matthew D. Martens[1,8], Nivedita Seshadri[2,8], Lucas Nguyen[8], Donald Chapman[8], Elizabeth S. Henson[3,10], Bo Xiang[4,8], Landon Falk[2,9], Arielys Mendoza[12], Sunil Rattan[4,11], Jared T. Field[1,8], Philip Kawalec[1,8], Spencer B. Gibson[3,10], Richard Keijzer[5,9], Ayesha Saleem[7,8], Grant M. Hatch[4,8], Christine A. Doucette[2,8], Jason M. Karch[12], Vernon W. Dolinsky[4,8], Ian M. Dixon[2,11], Adrian R. West[2,9], Christof Rampitsch[13], and Joseph W. Gordon[1,6,8,*]
>
>> Begin forwarded message:
>>
>> **From:** cddisease@springernature.com
>> **Subject:** **CDDIS-21-1957RRR Initial Quality Check**
>> **Date:** November 4, 2021 at 9:36:18 AM ADT
>> **To:** joseph.gordon@umanitoba.ca
>> **Reply-To:** cddisease@springernature.com
>>
>> **Caution:** This message was sent from outside the University of Manitoba.
>>
>> Dear Dr Gordon,
>>
>> In checking in your manuscript submitted to Cell Death & Disease it has come to our attention that the following must be addressed before we can proceed.
>>
>> 1. Please make sure you include a data availability statement in your Article file under its own subheading.
>>
>> 2. Please make sure all tables and figures are cited in the main text, in numerical order
>>
>> 3. It has come to our attention that your most recent author list differs from the one in your original submission.
>>
>> Please request agreement from all authors including additions and deletions, these can be collected in the following way:
>>
>> Email your co-authors with the change, and ask them to reply to your email confirming that they agree to these changes. Once you have collected these replies, please combine all of the co-authors' email responses in one document and upload this file to your submission.
>>
>> Your paper is now available for you to edit, you may access via the following link:
>>
>> https://mts-cddis.nature.com/cgi-bin/main.plex?el=A2CA6UZA6D2dOU5F1A9ftdqTfhMrqgyuhG60SwflYg7QZ
>>
>> [illegible]

(Press/Click on the above link to be automatically sent to the web page.)

Please make the correction(s) as specified above and resubmit your paper following the same steps as before.

If you have any questions please feel free to contact us.

Sincerely,

Editorial Office
Cell Death & Disease
cddisease@springernature.com

Our flexible approach during the COVID-19 pandemic
If you need more time at any stage of the peer-review process, please do let us know. While our systems will continue to remind you of the original timelines, we aim to be as flexible as possible during the current pandemic.

This email has been sent through the NPG Manuscript Tracking System NY-610A-NPG&MTS

Confidentiality Statement:

This e-mail is confidential and subject to copyright. Any unauthorised use or disclosure of its contents is prohibited. If you have received this email in error please notify our Manuscript Tracking System Helpdesk team at http://platformsupport.nature.com .

Details of the confidentiality and pre-publicity policy may be found here http://www.nature.com/authors/policies/confidentiality.html

Privacy Policy | Update Profile

From: **Ian Dixon** IDixon@sbrc.ca
Subject: RE: Fwd: CDDIS-21-1957RRR Initial Quality Check
Date: November 4, 2021 at 1:22 PM
To: Richard Keijzer richardkeijzer@gmail.com, Sunil Rattan SRattan@sbrc.ca, Ayesha Saleem ayesha.saleem@umanitoba.ca, landonfalk landonfalk@gmail.com, Nguyen Lucas nguyenlucas90@gmail.com, Matthew Martens marten22@myumanitoba.ca, Elizabeth Henson elizabeth.henson@umanitoba.ca, nivedi87@gmail.com, Christof Rampitsch chris.rampitsch@agr.gc.ca, Christine Doucette cdoucette@chrim.ca, Adrian West adrian.west@umanitoba.ca, Vern Dolinsky vernon.dolinsky@umanitoba.ca, Richard Keijzer rkeijzer@chrim.ca, Joe Gordon joseph.gordon@umanitoba.ca, Donald Chapman dchapman@chrim.ca, Spencer Gibson spencer.gibson@umanitoba.ca, Philip Kawalec kawalecp@myumanitoba.ca, Bo Xiang bxiang@chrim.ca, arielysm@bcm.edu, Grant Hatch ghatch@chrim.ca, Jason Karch jason.karch@bcm.edu, Jared Field umfiel26@myumanitoba.ca

---

****
Caution: This message was sent from outside the University of Manitoba.
****

Confirmed.

Kind regards,
Ian

____
From: Richard Keijzer [richardkeijzer@gmail.com]
Sent: November 4, 2021 11:03 AM
To: Sunil Rattan; Ayesha Saleem; landonfalk; Nguyen Lucas; Matthew Martens; Elizabeth Henson; nivedi87@gmail.com; Christof Rampitsch; Christine Doucette; Adrian West; Vern Dolinsky; Richard Keijzer; Joe Gordon; Ian Dixon; Donald Chapman; Spencer Gibson; Philip Kawalec; Bo Xiang; arielysm@bcm.edu; Grant Hatch; Jason Karch; Jared Field
Subject: Re: Fwd: CDDIS-21-1957RRR Initial Quality Check

Confirm

Thank you,

--
Richard Keijzer

On 4November, 2021 at 10:26:41, Joe Gordon (joseph.gordon@umanitoba.ca<mailto:joseph.gordon@umanitoba.ca>) wrote:

Hello everyone,

During the review process the author list for Matt's paper changed (see below). Most notably, Jared Field and Philip Kawalec were added for their assistance with the flow cytometry experiments.

The paper has been accepted, but the journal (Cell Death and Disease) needs confirmation that all authors agree to this authorship change.

Please reply as soon as you can with a simple "Confirm" and I will compile and upload.

Thank you for all your support.

Joe.

Matthew D. Martens1,8, Nivedita Seshadri2,8, Lucas Nguyen8, Donald Chapman8, Elizabeth S. Henson3,10, Bo Xiang4,8, Landon Falk2,9, Arielys Mendoza12, Sunil Rattan4,11, Jared T. Field1,8, Philip Kawalec1,8, Spencer B. Gibson3,10, Richard Keijzer5,9, Ayesha Saleem7,8, Grant M. Hatch4,8, Christine A. Doucette2,8, Jason M. Karch12, Vernon W. Dolinsky4,8, Ian M. Dixon2,11, Adrian R. West2,9, Christof Rampitsch13, and Joseph W. Gordon1,6,8,*

Begin forwarded message:

From: cddisease@springernature.com<mailto:cddisease@springernature.com>
Subject: CDDIS-21-1957RRR Initial Quality Check
Date: November 4, 2021 at 9:36:18 AM ADT
To: joseph.gordon@umanitoba.ca<mailto:joseph.gordon@umanitoba.ca>
Reply-To: cddisease@springernature.com<mailto:cddisease@springernature.com>

Caution: This message was sent from outside the University of Manitoba.
Dear Dr Gordon,

In checking in your manuscript submitted to Cell Death & Disease it has come to our attention that the following must be addressed before we can proceed.

1. Please make sure you include a data availability statement in your Article file under its own subheading

1. Please make sure you include a data availability statement in your Article file under its own subheading.

2. Please make sure all tables and figures are cited in the main text, in numerical order

3. It has come to our attention that your most recent author list differs from the one in your original submission.

Please request agreement from all authors including additions and deletions, these can be collected in the following way:

Email your co-authors with the change, and ask them to reply to your email confirming that they agree to these changes. Once you have collected these replies, please combine all of the co-authors' email responses in one document and upload this file to your submission.

Your paper is now available for you to edit, you may access via the following link:

https://mts-cddis.nature.com/cgi-bin/main.plex?el=A2CA6UZA6D2dOU5F1A9ftdqTfhMrqgyuhG60SwflYg7QZ

(Press/Click on the above link to be automatically sent to the web page.)

Please make the correction(s) as specified above and resubmit your paper following the same steps as before.

If you have any questions please feel free to contact us.

Sincerely,

Editorial Office
Cell Death & Disease
cddisease@springernature.com<mailto:cddisease@springernature.com>

Our flexible approach during the COVID-19 pandemic
If you need more time at any stage of the peer-review process, please do let us know. While our systems will continue to remind you of the original timelines, we aim to be as flexible as possible during the current pandemic.

This email has been sent through the NPG Manuscript Tracking System NY-610A-NPG&MTS

Confidentiality Statement:

This e-mail is confidential and subject to copyright. Any unauthorised use or disclosure of its contents is prohibited. If you have received this email in error please notify our Manuscript Tracking System Helpdesk team at <http://platformsupport.nature.com/> http://platformsupport.nature.com<http://platformsupport.nature.com/> .

Details of the confidentiality and pre-publicity policy may be found here <http://www.nature.com/authors/policies/confidentiality.html> http://www.nature.com/authors/policies/confidentiality.html

Privacy Policy<http://www.nature.com/info/privacy.html> | Update Profile<https://mts-cddis.nature.com/>

From: **Jared Field** umfiel26@myumanitoba.ca
Subject: Re: CDDIS-21-1957RRR Initial Quality Check
Date: November 4, 2021 at 1:33 PM
To: Joe Gordon Joseph.Gordon@umanitoba.ca

JF

Confirm.

Thank you,

Jared Field

PhD Student
Department of Human Anatomy & Cell Science
University of Manitoba
umfiel26@myumanitoba.ca

> On Nov 4, 2021, at 10:26 AM, Joe Gordon <Joseph.Gordon@umanitoba.ca> wrote:
>
> Hello everyone,
>
> During the review process the author list for Matt's paper changed (see below). Most notably, Jared Field and Philip Kawalec were added for their assistance with the flow cytometry experiments.
>
> The paper has been accepted, but the journal (Cell Death and Disease) needs confirmation that all authors agree to this authorship change.
>
> Please reply as soon as you can with a simple "Confirm" and I will compile and upload.
>
> Thank you for all your support.
>
> Joe.
>
> Matthew D. Martens[1,8], Nivedita Seshadri[2,8], Lucas Nguyen[8], Donald Chapman[8], Elizabeth S. Henson[3,10], Bo Xiang[4,8], Landon Falk[2,9], Arielys Mendoza[12], Sunil Rattan[4,11], Jared T. Field[1,8], Philip Kawalec[1,8], Spencer B. Gibson[3,10], Richard Keijzer[5,9], Ayesha Saleem[7,8], Grant M. Hatch[4,8], Christine A. Doucette[2,8], Jason M. Karch[12], Vernon W. Dolinsky[4,8], Ian M. Dixon[2,11], Adrian R. West[2,9], Christof Rampitsch[13], and Joseph W. Gordon[1,6,8,*]
>
>> Begin forwarded message:
>>
>> **From:** cddisease@springernature.com
>> **Subject: CDDIS-21-1957RRR Initial Quality Check**
>> **Date:** November 4, 2021 at 9:36:18 AM ADT
>> **To:** joseph.gordon@umanitoba.ca
>> **Reply-To:** cddisease@springernature.com
>>
>> **Caution:** This message was sent from outside the University of Manitoba.
>>
>> Dear Dr Gordon,
>>
>> In checking in your manuscript submitted to Cell Death & Disease it has come to our attention that the following must be addressed before we can proceed.
>>
>> 1. Please make sure you include a data availability statement in your Article file under its own subheading.
>>
>> 2. Please make sure all tables and figures are cited in the main text, in numerical order
>>
>> 3. It has come to our attention that your most recent author list differs from the one in your original submission.
>>
>> Please request agreement from all authors including additions and deletions, these can be collected in the following way:
>>
>> Email your co-authors with the change, and ask them to reply to your email confirming that they agree to these changes. Once you have collected these replies, please combine all of the co-authors' email responses in one document and upload this file to [illegible]

your submission.

Your paper is now available for you to edit, you may access via the following link:

https://mts-cddis.nature.com/cgi-bin/main.plex?el=A2CA6UZA6D2dOU5F1A9ftdqTfhMrqgyuhG60SwflYg7QZ

(Press/Click on the above link to be automatically sent to the web page.)

Please make the correction(s) as specified above and resubmit your paper following the same steps as before.

If you have any questions please feel free to contact us.

Sincerely,

Editorial Office
Cell Death & Disease
cddisease@springernature.com

Our flexible approach during the COVID-19 pandemic
If you need more time at any stage of the peer-review process, please do let us know. While our systems will continue to remind you of the original timelines, we aim to be as flexible as possible during the current pandemic.

This email has been sent through the NPG Manuscript Tracking System NY-610A-NPG&MTS

Confidentiality Statement:

This e-mail is confidential and subject to copyright. Any unauthorised use or disclosure of its contents is prohibited. If you have received this email in error please notify our Manuscript Tracking System Helpdesk team at http://platformsupport.nature.com .

Details of the confidentiality and pre-publicity policy may be found here http://www.nature.com/authors/policies/confidentiality.html

Privacy Policy | Update Profile

From: **Karch, Jason M.** Jason.Karch@bcm.edu
Subject: RE: CDDIS-21-1957RRR Initial Quality Check
Date: November 4, 2021 at 12:32 PM
To: Joe Gordon Joseph.Gordon@umanitoba.ca

JK

Caution: This message was sent from outside the University of Manitoba.

Confirm

From: Joe Gordon <Joseph.Gordon@umanitoba.ca>
Sent: Thursday, November 4, 2021 10:27 AM
To: Matthew Martens <marten22@myumanitoba.ca>; nivedi87@gmail.com; Nguyen Lucas <nguyenlucas90@gmail.com>; Donald Chapman <DChapman@chrim.ca>; Elizabeth Henson <Elizabeth.Henson@umanitoba.ca>; Bo Xiang <BXiang@chrim.ca>; landonfalk <landonfalk@gmail.com>; Mendoza, Arielys Melissa <Arielys.Mendoza@bcm.edu>; Sunil Rattan <SRattan@sbrc.ca>; Jared Field <umfiel26@myumanitoba.ca>; Philip Kawalec <kawalecp@myumanitoba.ca>; Spencer Gibson <Spencer.Gibson@umanitoba.ca>; Richard Keijzer <RKeijzer@chrim.ca>; Ayesha Saleem <Ayesha.Saleem@umanitoba.ca>; Grant Hatch <GHatch@chrim.ca>; Christine Doucette <CDoucette@chrim.ca>; Karch, Jason M. <Jason.Karch@bcm.edu>; Vern Dolinsky <Vernon.Dolinsky@umanitoba.ca>; Ian Dixon <idixon@sbrc.ca>; Adrian West <Adrian.West@umanitoba.ca>; Christof Rampitsch <chris.rampitsch@agr.gc.ca>
Subject: Fwd: CDDIS-21-1957RRR Initial Quality Check
Importance: High

*****CAUTION:*** This email is not from a BCM Source. Only click links or open attachments you know are safe.**

Hello everyone,

During the review process the author list for Matt’s paper changed (see below). Most notably, Jared Field and Philip Kawalec were added for their assistance with the flow cytometry experiments.

The paper has been accepted, but the journal (Cell Death and Disease) needs confirmation that all authors agree to this authorship change.

Please reply as soon as you can with a simple “Confirm” and I will compile and upload.

Thank you for all your support.

Joe.

Matthew D. Martens[1,8], Nivedita Seshadri[2,8], Lucas Nguyen[8], Donald Chapman[8], Elizabeth S. Henson[3,10], Bo Xiang[4,8], Landon Falk[2,9], Arielys Mendoza[12], Sunil Rattan[4,11], Jared T. Field[1,8], Philip Kawalec[1,8], Spencer B. Gibson[3,10], Richard Keijzer[5,9], Ayesha Saleem[7,8], Grant M. Hatch[4,8], Christine A. Doucette[2,8], Jason M. Karch[12], Vernon W. Dolinsky[4,8], Ian

[illegible]

M. Dixon[2,11], Adrian R. West[2,9], Christof Rampitsch[13], and Joseph W. Gordon[1,6,8,*]

Begin forwarded message:

From: cddisease@springernature.com
Subject: CDDIS-21-1957RRR Initial Quality Check
Date: November 4, 2021 at 9:36:18 AM ADT
To: joseph.gordon@umanitoba.ca
Reply-To: cddisease@springernature.com

Caution: This message was sent from outside the University of Manitoba.

Dear Dr Gordon,

In checking in your manuscript submitted to Cell Death & Disease it has come to our attention that the following must be addressed before we can proceed.

1. Please make sure you include a data availability statement in your Article file under its own subheading.

2. Please make sure all tables and figures are cited in the main text, in numerical order

3. It has come to our attention that your most recent author list differs from the one in your original submission.

Please request agreement from all authors including additions and deletions, these can be collected in the following way:

Email your co-authors with the change, and ask them to reply to your email confirming that they agree to these changes. Once you have collected these replies, please combine all of the co-authors' email responses in one document and upload this file to your submission.

Your paper is now available for you to edit, you may access via the following link:

https://mts-cddis.nature.com/cgi-bin/main.plex?el=A2CA6UZA6D2dOU5F1A9ftdqTfhMrqgyuhG60SwflYg7QZ

(Press/Click on the above link to be automatically sent to the web page.)

Please make the correction(s) as specified above and resubmit your paper following the same steps as before.

If you have any questions please feel free to contact us.

Sincerely,

Editorial Office
Cell Death & Disease
cddisease@springernature.com

Our flexible approach during the COVID-19 pandemic
If you need more time at any stage of the peer-review process, please do let us know. While our systems will continue to remind you of the original timelines, we aim to be as flexible as possible during the current pandemic.

This email has been sent through the NPG Manuscript Tracking System NY-610A-NPG&MTS

Confidentiality Statement:

This e-mail is confidential and subject to copyright. Any unauthorised use or disclosure of its contents is prohibited. If you have received this email in error please notify our Manuscript Tracking System Helpdesk team at http://platformsupport.nature.com .
Details of the confidentiality and pre-publicity policy may be found here http://www.nature.com/authors/policies/confidentiality.html
Privacy Policy | Update Profile

From: **Landon Falk** landonfalk@gmail.com
Subject: Re: CDDIS-21-1957RRR Initial Quality Check
Date: November 4, 2021 at 1:10 PM
To: Richard Keijzer richardkeijzer@gmail.com
Cc: Sunil Rattan SRattan@sbrc.ca, Ayesha Saleem ayesha.saleem@umanitoba.ca, Nguyen Lucas nguyenlucas90@gmail.com, Matthew Martens marten22@myumanitoba.ca, Elizabeth Henson Elizabeth.Henson@umanitoba.ca, nivedi87@gmail.com, Christof Rampitsch chris.rampitsch@agr.gc.ca, Christine Doucette CDoucette@chrim.ca, Adrian West Adrian.West@umanitoba.ca , Vern Dolinsky Vernon.Dolinsky@umanitoba.ca, Richard Keijzer RKeijzer@chrim.ca, Joe Gordon Joseph.Gordon@umanitoba.ca , Ian Dixon IDixon@sbrc.ca, Donald Chapman DChapman@chrim.ca, Spencer Gibson Spencer.Gibson@umanitoba.ca, Philip Kawalec kawalecp@myumanitoba.ca, Bo Xiang BXiang@chrim.ca, arielysm@bcm.edu, Grant Hatch GHatch@chrim.ca, Jason Karch Jason.karch@bcm.edu, Jared Field umfiel26@myumanitoba.ca

LF

Caution: This message was sent from outside the University of Manitoba.

Confirm, congratulations on a wonderful paper!
Landon

> On Nov 4, 2021, at 11:03 AM, Richard Keijzer <richardkeijzer@gmail.com> wrote:
>
> Confirm
>
> Thank you,
>
> --
> Richard Keijzer
>
> On 4November, 2021 at 10:26:41, Joe Gordon (joseph.gordon@umanitoba.ca) wrote:
>
>> Hello everyone,
>>
>> During the review process the author list for Matt's paper changed (see below). Most notably, Jared Field and Philip Kawalec were added for their assistance with the flow cytometry experiments.
>>
>> The paper has been accepted, but the journal (Cell Death and Disease) needs confirmation that all authors agree to this authorship change.
>>
>> Please reply as soon as you can with a simple "Confirm" and I will compile and upload.
>>
>> Thank you for all your support.
>>
>> Joe.
>>
>> Matthew D. Martens[1,8], Nivedita Seshadri[2,8], Lucas Nguyen[8], Donald Chapman[8], Elizabeth S. Henson[3,10], Bo Xiang[4,8], Landon Falk[2,9], Arielys Mendoza[12], Sunil Rattan[4,11], Jared T. Field[1,8], Philip Kawalec[1,8], Spencer B. Gibson[3,10], Richard Keijzer[5,9], Ayesha Saleem[7,8], Grant M. Hatch[4,8], Christine A. Doucette[2,8], Jason M. Karch[12], Vernon W. Dolinsky[4,8], Ian M. Dixon[2,11], Adrian R. West[2,9], Christof Rampitsch[13], and Joseph W. Gordon[1,6,8,*]
>>
>>> Begin forwarded message:

From: cddisease@springernature.com
Subject: CDDIS-21-1957RRR Initial Quality Check
Date: November 4, 2021 at 9:36:18 AM ADT
To: joseph.gordon@umanitoba.ca
Reply-To: cddisease@springernature.com

Caution: This message was sent from outside the University of Manitoba.

Dear Dr Gordon,

In checking in your manuscript submitted to Cell Death & Disease it has come to our attention that the following must be addressed before we can proceed.

1. Please make sure you include a data availability statement in your Article file under its own subheading.

2. Please make sure all tables and figures are cited in the main text, in numerical order

3. It has come to our attention that your most recent author list differs from the one in your original submission.

Please request agreement from all authors including additions and deletions, these can be collected in the following way:

Email your co-authors with the change, and ask them to reply to your email confirming that they agree to these changes. Once you have collected these replies, please combine all of the co-authors' email responses in one document and upload this file to your submission.

Your paper is now available for you to edit, you may access via the following link:

https://mts-cddis.nature.com/cgi-bin/main.plex?el=A2CA6UZA6D2dOU5F1A9ftdqTfhMrqgyuhG60SwflYg7QZ

(Press/Click on the above link to be automatically sent to the web page.)

Please make the correction(s) as specified above and resubmit your paper following the same steps as before.

If you have any questions please feel free to contact us.

Sincerely,

Editorial Office
Cell Death & Disease
cddisease@springernature.com

Our flexible approach during the COVID-19 pandemic
If you need more time at any stage of the peer-review process, please do let us know. While our systems will continue to remind you of the original timelines, we aim to be as flexible as possible during the current pandemic.

This email has been sent through the NPG Manuscript Tracking System NY-610A-NPG&MTS

Confidentiality Statement:

This e-mail is confidential and subject to copyright. Any unauthorised use or disclosure of its contents is prohibited. If you have received this email in error please notify our Manuscript Tracking System Helpdesk team at http://platformsupport.nature.com .

Details of the confidentiality and pre-publicity policy may be found here http://www.nature.com/authors/policies/confidentiality.html

Privacy Policy | Update Profile

From: **Elizabeth Henson** Elizabeth.Henson@umanitoba.ca
Subject: Re: CDDIS-21-1957RRR Initial Quality Check
Date: November 4, 2021 at 1:06 PM
To: Joe Gordon Joseph.Gordon@umanitoba.ca

EH

Confirm.

And congratulations!!!

> On Nov 4, 2021, at 10:26 AM, Joe Gordon <Joseph.Gordon@umanitoba.ca> wrote:
>
> Hello everyone,
>
> During the review process the author list for Matt's paper changed (see below). Most notably, Jared Field and Philip Kawalec were added for their assistance with the flow cytometry experiments.
>
> The paper has been accepted, but the journal (Cell Death and Disease) needs confirmation that all authors agree to this authorship change.
>
> Please reply as soon as you can with a simple "Confirm" and I will compile and upload.
>
> Thank you for all your support.
>
> Joe.
>
> Matthew D. Martens[1,8], Nivedita Seshadri[2,8], Lucas Nguyen[8], Donald Chapman[8], Elizabeth S. Henson[3,10], Bo Xiang[4,8], Landon Falk[2,9], Arielys Mendoza[12], Sunil Rattan[4,11], Jared T. Field[1,8], Philip Kawalec[1,8], Spencer B. Gibson[3,10], Richard Keijzer[5,9], Ayesha Saleem[7,8], Grant M. Hatch[4,8], Christine A. Doucette[2,8], Jason M. Karch[12], Vernon W. Dolinsky[4,8], Ian M. Dixon[2,11], Adrian R. West[2,9], Christof Rampitsch[13], and Joseph W. Gordon[1,6,8,*]
>
>> Begin forwarded message:
>>
>> **From:** cddisease@springernature.com
>> **Subject: CDDIS-21-1957RRR Initial Quality Check**
>> **Date:** November 4, 2021 at 9:36:18 AM ADT
>> **To:** joseph.gordon@umanitoba.ca
>> **Reply-To:** cddisease@springernature.com
>>
>> **Caution:** This message was sent from outside the University of Manitoba.
>>
>> Dear Dr Gordon,
>>
>> In checking in your manuscript submitted to Cell Death & Disease it has come to our attention that the following must be addressed before we can proceed.
>>
>> 1. Please make sure you include a data availability statement in your Article file under its own subheading.
>>
>> 2. Please make sure all tables and figures are cited in the main text, in numerical order
>>
>> 3. It has come to our attention that your most recent author list differs from the one in your original submission.
>>
>> Please request agreement from all authors including additions and deletions, these can be collected in the following way:
>>
>> Email your co-authors with the change, and ask them to reply to your email confirming that they agree to these changes. Once you have collected these replies, please combine all of the co-authors' email responses in one document and upload this file to your submission.
>>
>> Your paper is now available for you to edit, you may access via the following link:
>>
>> https://mts-cddis.nature.com/cgi-bin/main.plex?el=A2CA6UZA6D2dOU5F1A9ftdqTfhMrqgyuhG60SwflYg7QZ
>>
>> (Press/Click on the above link to be automatically sent to the web page.)
>>
>> Please make the correction(s) as specified above and resubmit your paper following the same steps as before.

If you have any questions please feel free to contact us.

Sincerely,

Editorial Office
Cell Death & Disease
cddisease@springernature.com

Our flexible approach during the COVID-19 pandemic
If you need more time at any stage of the peer-review process, please do let us know. While our systems will continue to remind you of the original timelines, we aim to be as flexible as possible during the current pandemic.

This email has been sent through the NPG Manuscript Tracking System NY-610A-NPG&MTS

Confidentiality Statement:

This e-mail is confidential and subject to copyright. Any unauthorised use or disclosure of its contents is prohibited. If you have received this email in error please notify our Manuscript Tracking System Helpdesk team at http://platformsupport.nature.com .

Details of the confidentiality and pre-publicity policy may be found here http://www.nature.com/authors/policies/confidentiality.html

Privacy Policy | Update Profile

Lucas Nguyen <nguyenlucas90@gmail.com> wrote:

Hey Joe,

Great to hear from you! I would be happy to be co-author on this paper. The figures look great!

Hope all is well with you too!

Regards,

Lucas

Sent from my iPhone

From: **Matthew Martens** marten22@myumanitoba.ca
Subject: Re: CDDIS-21-1957RRR Initial Quality Check
Date: November 4, 2021 at 12:28 PM
To: Joe Gordon Joseph.Gordon@umanitoba.ca

Confirm

> On Nov 4, 2021, at 9:26 AM, Joe Gordon <Joseph.Gordon@umanitoba.ca> wrote:
>
> Hello everyone,
>
> During the review process the author list for Matt's paper changed (see below). Most notably, Jared Field and Philip Kawalec were added for their assistance with the flow cytometry experiments.
>
> The paper has been accepted, but the journal (Cell Death and Disease) needs confirmation that all authors agree to this authorship change.
>
> Please reply as soon as you can with a simple "Confirm" and I will compile and upload.
>
> Thank you for all your support.
>
> Joe.
>
> Matthew D. Martens[1,8], Nivedita Seshadri[2,8], Lucas Nguyen[8], Donald Chapman[8], Elizabeth S. Henson[3,10], Bo Xiang[4,8], Landon Falk[2,9], Arielys Mendoza[12], Sunil Rattan[4,11], Jared T. Field[1,8], Philip Kawalec[1,8], Spencer B. Gibson[3,10], Richard Keijzer[5,9], Ayesha Saleem[7,8], Grant M. Hatch[4,8], Christine A. Doucette[2,8], Jason M. Karch[12], Vernon W. Dolinsky[4,8], Ian M. Dixon[2,11], Adrian R. West[2,9], Christof Rampitsch[13], and Joseph W. Gordon[1,6,8,*]
>
> > Begin forwarded message:
> >
> > **From:** cddisease@springernature.com
> > **Subject: CDDIS-21-1957RRR Initial Quality Check**
> > **Date:** November 4, 2021 at 9:36:18 AM ADT
> > **To:** joseph.gordon@umanitoba.ca
> > **Reply-To:** cddisease@springernature.com
> >
> > **Caution:** This message was sent from outside the University of Manitoba.
> >
> > Dear Dr Gordon,
> >
> > In checking in your manuscript submitted to Cell Death & Disease it has come to our attention that the following must be addressed before we can proceed.
> >
> > 1. Please make sure you include a data availability statement in your Article file under its own subheading.
> >
> > 2. Please make sure all tables and figures are cited in the main text, in numerical order
> >
> > 3. It has come to our attention that your most recent author list differs from the one in your original submission.
> >
> > Please request agreement from all authors including additions and deletions, these can be collected in the following way:
> >
> > Email your co-authors with the change, and ask them to reply to your email confirming that they agree to these changes. Once you have collected these replies, please combine all of the co-authors' email responses in one document and upload this file to your submission.
> >
> > Your paper is now available for you to edit, you may access via the following link:
> >
> > https://mts-cddis.nature.com/cgi-bin/main.plex?el=A2CA6UZA6D2dOU5F1A9ftdqTfhMrqgyuhG60SwflYg7QZ
> >
> > (Press/Click on the above link to be automatically sent to the web page.)
> >
> > Please make the correction(s) as specified above and resubmit your paper following the same steps as before.
> >
> > If you have any questions please feel free to contact us.
> >
> > [illegible]

Sincerely,

Editorial Office
Cell Death & Disease
cddisease@springernature.com

Our flexible approach during the COVID-19 pandemic
If you need more time at any stage of the peer-review process, please do let us know. While our systems will continue to remind you of the original timelines, we aim to be as flexible as possible during the current pandemic.

This email has been sent through the NPG Manuscript Tracking System NY-610A-NPG&MTS

Confidentiality Statement:

This e-mail is confidential and subject to copyright. Any unauthorised use or disclosure of its contents is prohibited. If you have received this email in error please notify our Manuscript Tracking System Helpdesk team at http://platformsupport.nature.com .

Details of the confidentiality and pre-publicity policy may be found here http://www.nature.com/authors/policies/confidentiality.html

Privacy Policy | Update Profile

From: **nivedita seshadri** nivedi87@gmail.com
Subject: Re: CDDIS-21-1957RRR Initial Quality Check
Date: November 4, 2021 at 12:38 PM
To: Joe Gordon Joseph.Gordon@umanitoba.ca

Caution: This message was sent from outside the University of Manitoba.

Confirm

Thank you
Nivedita

On Thu, Nov 4, 2021 at 11:26 AM Joe Gordon <Joseph.Gordon@umanitoba.ca> wrote:

> Hello everyone,
>
> During the review process the author list for Matt's paper changed (see below). Most notably, Jared Field and Philip Kawalec were added for their assistance with the flow cytometry experiments.
>
> The paper has been accepted, but the journal (Cell Death and Disease) needs confirmation that all authors agree to this authorship change.
>
> Please reply as soon as you can with a simple "Confirm" and I will compile and upload.
>
> Thank you for all your support.
>
> Joe.
>
> Matthew D. Martens[1,8], Nivedita Seshadri[2,8], Lucas Nguyen[8], Donald Chapman[8], Elizabeth S. Henson[3,10], Bo Xiang[4,8], Landon Falk[2,9], Arielys Mendoza[12], Sunil Rattan[4,11], Jared T. Field[1,8], Philip Kawalec[1,8], Spencer B. Gibson[3,10], Richard Keijzer[5,9], Ayesha Saleem[7,8], Grant M. Hatch[4,8], Christine A. Doucette[2,8], Jason M. Karch[12], Vernon W. Dolinsky[4,8], Ian M. Dixon[2,11], Adrian R. West[2,9], Christof Rampitsch[13], and Joseph W. Gordon[1,6,8,*]
>
> > Begin forwarded message:
> >
> > **From:** cddisease@springernature.com
> > **Subject:** **CDDIS-21-1957RRR Initial Quality Check**
> > **Date:** November 4, 2021 at 9:36:18 AM ADT
> > **To:** joseph.gordon@umanitoba.ca
> > **Reply-To:** cddisease@springernature.com
> >
> > **Caution:** This message was sent from outside the University of Manitoba.
> >
> > Dear Dr Gordon,
> >
> > In checking in your manuscript submitted to Cell Death & Disease it has come to our attention that the following must be addressed before we can proceed.
> >
> > 1. Please make sure you include a data availability statement in your Article file under its own subheading.
> >
> > 2. Please make sure all tables and figures are cited in the main text, in numerical order
> >
> > 3. It has come to our attention that your most recent author list differs from the one in your original submission.
> >
> > Please request agreement from all authors including additions and deletions, these can be collected in the following way:
> >
> > Email your co-authors with the change, and ask them to reply to your email confirming that they agree to these changes. Once you have collected these replies, please combine all of the co-authors' email responses in one document and upload this file to your submission.
> >
> > Your paper is now available for you to edit, you may access via the following link:
> >
> > https://mts-cddis.nature.com/cgi-bin/main.plex?el=A2CA6UZA6D2dOU5F1A9ftdqTfhMrqgyuhG60SwflYg7QZ
> >
> > (Press/Click on the above link to be automatically sent to the web page.)
> >
> > Please make the correction(s) as specified above and resubmit your paper following the same steps as before

Please make the correction(s) as specified above and resubmit your paper following the same steps as before.

If you have any questions please feel free to contact us.

Sincerely,

Editorial Office
Cell Death & Disease
cddisease@springernature.com

Our flexible approach during the COVID-19 pandemic
If you need more time at any stage of the peer-review process, please do let us know. While our systems will continue to remind you of the original timelines, we aim to be as flexible as possible during the current pandemic.

This email has been sent through the NPG Manuscript Tracking System NY-610A-NPG&MTS

Confidentiality Statement:

This e-mail is confidential and subject to copyright. Any unauthorised use or disclosure of its contents is prohibited. If you have received this email in error please notify our Manuscript Tracking System Helpdesk team at http://platformsupport.nature.com .

Details of the confidentiality and pre-publicity policy may be found here http://www.nature.com/authors/policies/confidentiality.html

Privacy Policy | Update Profile

--
Nivedita Seshadri
Graduate student (PhD Candidate)
Dr. Christine Doucette's Lab
608, John Buhler Research Ctr
745, Bannnatyne Avenue
Winnipeg, MB, CANADA
R3E 0J9

From: **Philip Kawalec** kawalecp@myumanitoba.ca
Subject: Re: CDDIS-21-1957RRR Initial Quality Check
Date: November 4, 2021 at 12:48 PM
To: Joe Gordon Joseph.Gordon@umanitoba.ca

PK

Confirm! Thanks Joe!

Philip Kawalec
Medical Student | Class of 2023
Max Rady College of Medicine

From: Joe Gordon <Joseph.Gordon@umanitoba.ca>
Sent: Thursday, November 4, 2021, 10:26
To: Matthew Martens; nivedi87@gmail.com; Nguyen Lucas; Donald Chapman; Elizabeth Henson; Bo Xiang; landonfalk; arielysm@bcm.edu; Sunil Rattan; Jared Field; Philip Kawalec; Spencer Gibson; Richard Keijzer; Ayesha Saleem; Grant Hatch; Christine Doucette; Jason Karch; Vern Dolinsky; Ian Dixon; Adrian West; Christof Rampitsch
Subject: Fwd: CDDIS-21-1957RRR Initial Quality Check

Hello everyone,

During the review process the author list for Matt's paper changed (see below). Most notably, Jared Field and Philip Kawalec were added for their assistance with the flow cytometry experiments.

The paper has been accepted, but the journal (Cell Death and Disease) needs confirmation that all authors agree to this authorship change.

Please reply as soon as you can with a simple "Confirm" and I will compile and upload.

Thank you for all your support.

Joe.

Matthew D. Martens[1,8], Nivedita Seshadri[2,8], Lucas Nguyen[8], Donald Chapman[8], Elizabeth S. Henson[3,10], Bo Xiang[4,8], Landon Falk[2,9], Arielys Mendoza[12], Sunil Rattan[4,11], Jared T. Field[1,8], Philip Kawalec[1,8], Spencer B. Gibson[3,10], Richard Keijzer[5,9], Ayesha Saleem[7,8], Grant M. Hatch[4,8], Christine A. Doucette[2,8], Jason M. Karch[12], Vernon W. Dolinsky[4,8], Ian M. Dixon[2,11], Adrian R. West[2,9], Christof Rampitsch[13], and Joseph W. Gordon[1,6,8,*]

> Begin forwarded message:
>
> **From:** cddisease@springernature.com
> **Subject:** **CDDIS-21-1957RRR Initial Quality Check**
> **Date:** November 4, 2021 at 9:36:18 AM ADT
> **To:** joseph.gordon@umanitoba.ca
> **Reply-To:** cddisease@springernature.com
>
> **Caution:** This message was sent from outside the University of Manitoba.
>
> Dear Dr Gordon,
>
> In checking in your manuscript submitted to Cell Death & Disease it has come to our attention that the following must be addressed before we can proceed.
>
> 1. Please make sure you include a data availability statement in your Article file under its own subheading.
>
> 2. Please make sure all tables and figures are cited in the main text, in numerical order
>
> 3. It has come to our attention that your most recent author list differs from the one in your original submission.
>
> Please request agreement from all authors including additions and deletions, these can be collected in the following way:
>
> Email your co-authors with the change, and ask them to reply to your email confirming that they agree to these changes. Once you have collected these replies, please combine all of the co-authors' email responses in one document and upload this file to your submission.

Your paper is now available for you to edit, you may access via the following link:

https://mts-cddis.nature.com/cgi-bin/main.plex?el=A2CA6UZA6D2dOU5F1A9ftdqTfhMrqgyuhG60SwflYg7QZ

(Press/Click on the above link to be automatically sent to the web page.)

Please make the correction(s) as specified above and resubmit your paper following the same steps as before.

If you have any questions please feel free to contact us.

Sincerely,

Editorial Office
Cell Death & Disease
cddisease@springernature.com

Our flexible approach during the COVID-19 pandemic
If you need more time at any stage of the peer-review process, please do let us know. While our systems will continue to remind you of the original timelines, we aim to be as flexible as possible during the current pandemic.

This email has been sent through the NPG Manuscript Tracking System NY-610A-NPG&MTS

Confidentiality Statement:

This e-mail is confidential and subject to copyright. Any unauthorised use or disclosure of its contents is prohibited. If you have received this email in error please notify our Manuscript Tracking System Helpdesk team at http://platformsupport.nature.com .

Details of the confidentiality and pre-publicity policy may be found here http://www.nature.com/authors/policies/confidentiality.html

Privacy Policy | Update Profile

From: Richard Keijzer richardkeijzer@gmail.com
Subject: Re: Fwd: CDDIS-21-1957RRR Initial Quality Check
Date: November 4, 2021 at 1:04 PM
To: Sunil Rattan srattan@sbrc.ca, Ayesha Saleem ayesha.saleem@umanitoba.ca, landonfalk landonfalk@gmail.com, Nguyen Lucas nguyenlucas90@gmail.com, Matthew Martens marten22@myumanitoba.ca, Elizabeth Henson elizabeth.henson@umanitoba.ca, nivedi87@gmail.com, Christof Rampitsch chris.rampitsch@agr.gc.ca, Christine Doucette cdoucette@chrim.ca, Adrian West adrian.west@umanitoba.ca, Vern Dolinsky vernon.dolinsky@umanitoba.ca, Richard Keijzer rkeijzer@chrim.ca, Joe Gordon joseph.gordon@umanitoba.ca, Ian Dixon idixon@sbrc.ca, Donald Chapman dchapman@chrim.ca, Spencer Gibson spencer.gibson@umanitoba.ca, Philip Kawalec kawalecp@myumanitoba.ca, Bo Xiang bxiang@chrim.ca, arielysm@bcm.edu, Grant Hatch ghatch@chrim.ca, Jason Karch jason.karch@bcm.edu, Jared Field umfiel26@myumanitoba.ca

RK

Caution: This message was sent from outside the University of Manitoba.

Confirm

Thank you,

--
Richard Keijzer

On 4November, 2021 at 10:26:41, Joe Gordon (joseph.gordon@umanitoba.ca) wrote:

> Hello everyone,
>
> During the review process the author list for Matt's paper changed (see below). Most notably, Jared Field and Philip Kawalec were added for their assistance with the flow cytometry experiments.
>
> The paper has been accepted, but the journal (Cell Death and Disease) needs confirmation that all authors agree to this authorship change.
>
> Please reply as soon as you can with a simple "Confirm" and I will compile and upload.
>
> Thank you for all your support.
>
> Joe.
>
> Matthew D. Martens[1,8], Nivedita Seshadri[2,8], Lucas Nguyen[8], Donald Chapman[8], Elizabeth S. Henson[3,10], Bo Xiang[4,8], Landon Falk[2,9], Arielys Mendoza[12], Sunil Rattan[4,11], Jared T. Field[1,8], Philip Kawalec[1,8], Spencer B. Gibson[3,10], Richard Keijzer[5,9], Ayesha Saleem[7,8], Grant M. Hatch[4,8], Christine A. Doucette[2,8], Jason M. Karch[12], Vernon W. Dolinsky[4,8], Ian M. Dixon[2,11], Adrian R. West[2,9], Christof Rampitsch[13], and Joseph W. Gordon[1,6,8,*]
>
> > Begin forwarded message:
> >
> > **From:** cddisease@springernature.com
> > **Subject:** **CDDIS-21-1957RRR Initial Quality Check**
> > **Date:** November 4, 2021 at 9:36:18 AM ADT
> > **To:** joseph.gordon@umanitoba.ca
> > **Reply-To:** cddisease@springernature.com
> >
> > **Caution:** This message was sent from outside the University of Manitoba.

Dear Dr Gordon,

In checking in your manuscript submitted to Cell Death & Disease it has come to our attention that the following must be addressed before we can proceed.

1. Please make sure you include a data availability statement in your Article file under its own subheading.

2. Please make sure all tables and figures are cited in the main text, in numerical order

3. It has come to our attention that your most recent author list differs from the one in your original submission.

Please request agreement from all authors including additions and deletions, these can be collected in the following way:

Email your co-authors with the change, and ask them to reply to your email confirming that they agree to these changes. Once you have collected these replies, please combine all of the co-authors' email responses in one document and upload this file to your submission.

Your paper is now available for you to edit, you may access via the following link:

https://mts-cddis.nature.com/cgi-bin/main.plex?
el=A2CA6UZA6D2dOU5F1A9ftdqTfhMrqgyuhG60SwflYg7QZ

(Press/Click on the above link to be automatically sent to the web page.)

Please make the correction(s) as specified above and resubmit your paper following the same steps as before.

If you have any questions please feel free to contact us.

Sincerely,

Editorial Office
Cell Death & Disease
cddisease@springernature.com

Our flexible approach during the COVID-19 pandemic
If you need more time at any stage of the peer-review process, please do let us know. While our systems will continue to remind you of the original timelines, we aim to be as flexible as possible during the current pandemic.

This email has been sent through the NPG Manuscript Tracking System NY-610A-NPG&MTS

Confidentiality Statement:

This e-mail is confidential and subject to copyright. Any unauthorised use or disclosure of its contents is prohibited. If you have received this email in error please notify our Manuscript Tracking System Helpdesk team at http://platformsupport.nature.com .

Details of the confidentiality and pre-publicity policy may be found here
http://www.nature.com/authors/policies/confidentiality.html

Privacy Policy | Update Profile

From: Spencer Gibson Spencer.Gibson@umanitoba.ca
Subject: RE: CDDIS-21-1957RRR Initial Quality Check
Date: November 4, 2021 at 12:33 PM
To: Joe Gordon Joseph.Gordon@umanitoba.ca

SG

Confirm, I accept the authorship change.

Spencer Gibson, Ph.D.
Kipnes Endowed Chair in Lymphatic Disorders
Professor
Department of Oncology
University of Alberta

Adjunct Scientist/Professor
CancerCare Manitoba Research Institute
Department of Biochemistry and Medical Genetics
And Immunology
University of Manitoba

From: Joe Gordon <Joseph.Gordon@umanitoba.ca>
Sent: November 4, 2021 9:27 AM
To: Matthew Martens <marten22@myumanitoba.ca>; nivedi87@gmail.com; Nguyen Lucas <nguyenlucas90@gmail.com>; Donald Chapman <DChapman@chrim.ca>; Elizabeth Henson <Elizabeth.Henson@umanitoba.ca>; Bo Xiang <BXiang@chrim.ca>; landonfalk <landonfalk@gmail.com>; arielysm@bcm.edu; Sunil Rattan <SRattan@sbrc.ca>; Jared Field <umfiel26@myumanitoba.ca>; Philip Kawalec <kawalecp@myumanitoba.ca>; Spencer Gibson <Spencer.Gibson@umanitoba.ca>; Richard Keijzer <RKeijzer@chrim.ca>; Ayesha Saleem <Ayesha.Saleem@umanitoba.ca>; Grant Hatch <GHatch@chrim.ca>; Christine Doucette <CDoucette@chrim.ca>; Jason Karch <Jason.karch@bcm.edu>; Vern Dolinsky <Vernon.Dolinsky@umanitoba.ca>; Ian Dixon <idixon@sbrc.ca>; Adrian West <Adrian.West@umanitoba.ca>; Christof Rampitsch <chris.rampitsch@agr.gc.ca>
Subject: Fwd: CDDIS-21-1957RRR Initial Quality Check
Importance: High

Hello everyone,

During the review process the author list for Matt's paper changed (see below). Most notably, Jared Field and Philip Kawalec were added for their assistance with the flow cytometry experiments.

The paper has been accepted, but the journal (Cell Death and Disease) needs confirmation that all authors agree to this authorship change.

Please reply as soon as you can with a simple "Confirm" and I will compile and upload.

Thank you for all your support.

Joe.

Matthew D. Martens[1,8], Nivedita Seshadri[2,8], Lucas Nguyen[8], Donald Chapman[8], Elizabeth S

Matthew D. Martens[illegible], Nivedita Seshadri[illegible], Lucas Nguyen[illegible], Donald Chapman[illegible], Elizabeth S. Henson[3,10], Bo Xiang[4,8], Landon Falk[2,9], Arielys Mendoza[12], Sunil Rattan[4,11], Jared T. Field[1,8], Philip Kawalec[1,8], Spencer B. Gibson[3,10], Richard Keijzer[5,9], Ayesha Saleem[7,8], Grant M. Hatch[4,8], Christine A. Doucette[2,8], Jason M. Karch[12], Vernon W. Dolinsky[4,8], Ian M. Dixon[2,11], Adrian R. West[2,9], Christof Rampitsch[13], and Joseph W. Gordon[1,6,8,*]

Begin forwarded message:

From: cddisease@springernature.com
Subject: CDDIS-21-1957RRR Initial Quality Check
Date: November 4, 2021 at 9:36:18 AM ADT
To: joseph.gordon@umanitoba.ca
Reply-To: cddisease@springernature.com

Caution: This message was sent from outside the University of Manitoba.

Dear Dr Gordon,

In checking in your manuscript submitted to Cell Death & Disease it has come to our attention that the following must be addressed before we can proceed.

1. Please make sure you include a data availability statement in your Article file under its own subheading.

2. Please make sure all tables and figures are cited in the main text, in numerical order

3. It has come to our attention that your most recent author list differs from the one in your original submission.

Please request agreement from all authors including additions and deletions, these can be collected in the following way:

Email your co-authors with the change, and ask them to reply to your email confirming that they agree to these changes. Once you have collected these replies, please combine all of the co-authors' email responses in one document and upload this file to your submission.

Your paper is now available for you to edit, you may access via the following link:

https://mts-cddis.nature.com/cgi-bin/main.plex?el=A2CA6UZA6D2dOU5F1A9ftdqTfhMrqgyuhG60SwflYg7QZ

(Press/Click on the above link to be automatically sent to the web page.)

Please make the correction(s) as specified above and resubmit your paper following the same steps as before.

If you have any questions please feel free to contact us.

Sincerely,

Editorial Office
Cell Death & Disease
cddisease@springernature.com

Our flexible approach during the COVID-19 pandemic
If you need more time at any stage of the peer-review process, please do let us know. While our systems will continue to remind you of the original timelines, we aim to be as flexible as possible during the current pandemic.

This email has been sent through the NPG Manuscript Tracking System NY-610A-NPG&MTS

Confidentiality Statement:

This e-mail is confidential and subject to copyright. Any unauthorised use or disclosure of its contents is prohibited. If you have received this email in error please notify our Manuscript Tracking System Helpdesk team at http://platformsupport.nature.com .
Details of the confidentiality and pre-publicity policy may be found here http://www.nature.com/authors/policies/confidentiality.html
Privacy Policy | Update Profile

From: **Sunil Rattan** SRattan@sbrc.ca
Subject: RE: CDDIS-21-1957RRR Initial Quality Check
Date: November 4, 2021 at 1:07 PM
To: Joe Gordon Joseph.Gordon@umanitoba.ca

SR

Caution: This message was sent from outside the University of Manitoba.

Confirm.

Sunil Rattan

Technician/Lab Manager
Department of Physiology and Pathophysiology
Max Rady College of Medicine, Rady Faculty of Health Science
University of Manitoba
St. Boniface Hospital Albrechtsen Research Centre
Institute of Cardiovascular Sciences
Rm 3010 - 351 Taché Avenue
Winnipeg, Manitoba
Canada, R2H 2A6
☎Tel: 204.235.3171 Fax: 204.233.6723
Email: srattan@sbrc.ca / sunil.rattan@umanitoba.ca / srattan@sbgh.mb.ca

---

From: Joe Gordon <Joseph.Gordon@umanitoba.ca>
Sent: Thursday, November 4, 2021 10:27 AM
To: Matthew Martens <marten22@myumanitoba.ca>; nivedi87@gmail.com; Nguyen Lucas <nguyenlucas90@gmail.com>; Donald Chapman <DChapman@chrim.ca>; Elizabeth Henson <Elizabeth.Henson@umanitoba.ca>; Bo Xiang <BXiang@chrim.ca>; landonfalk <landonfalk@gmail.com>; arielysm@bcm.edu; Sunil Rattan <SRattan@sbrc.ca>; Jared Field <umfiel26@myumanitoba.ca>; Philip Kawalec <kawalecp@myumanitoba.ca>; Spencer Gibson <Spencer.Gibson@umanitoba.ca>; Richard Keijzer <RKeijzer@chrim.ca>; Ayesha Saleem <Ayesha.Saleem@umanitoba.ca>; Grant Hatch <GHatch@chrim.ca>; Christine Doucette <CDoucette@chrim.ca>; Jason Karch <Jason.karch@bcm.edu>; Vern Dolinsky <Vernon.Dolinsky@umanitoba.ca>; Ian Dixon <IDixon@sbrc.ca>; Adrian West <Adrian.West@umanitoba.ca>; Christof Rampitsch <chris.rampitsch@agr.gc.ca>
Subject: Fwd: CDDIS-21-1957RRR Initial Quality Check
Importance: High

Hello everyone,

During the review process the author list for Matt's paper changed (see below). Most notably, Jared Field and Philip Kawalec were added for their assistance with the flow cytometry experiments.

The paper has been accepted, but the journal (Cell Death and Disease) needs confirmation that all authors agree to this authorship change.

Please reply as soon as you can with a simple "Confirm" and I will compile and upload.

Thank you for all your support.

Joe.

Matthew D. Martens[1,8], Nivedita Seshadri[2,8], Lucas Nguyen[8], Donald Chapman[8], Elizabeth S. Henson[3,10], Bo Xiang[4,8], Landon Falk[2,9], Arielys Mendoza[12], Sunil Rattan[4,11], Jared T. Field[1,8], Philip Kawalec[1,8], Spencer B. Gibson[3,10], Richard Keijzer[5,9], Ayesha Saleem[7,8], Grant M. Hatch[4,8], Christine A. Doucette[2,8], Jason M. Karch[12], Vernon W. Dolinsky[4,8], Ian M. Dixon[2,11], Adrian R. West[2,9], Christof Rampitsch[13], and Joseph W. Gordon[1,6,8,*]

Begin forwarded message:

From: cddisease@springernature.com
Subject: CDDIS-21-1957RRR Initial Quality Check
Date: November 4, 2021 at 9:36:18 AM ADT
To: joseph.gordon@umanitoba.ca
Reply-To: cddisease@springernature.com

Caution: This message was sent from outside the University of Manitoba.

Dear Dr Gordon,

In checking in your manuscript submitted to Cell Death & Disease it has come to our attention that the following must be addressed before we can proceed.

1. Please make sure you include a data availability statement in your Article file under its own subheading.

2. Please make sure all tables and figures are cited in the main text, in numerical order

3. It has come to our attention that your most recent author list differs from the one in your original submission.

Please request agreement from all authors including additions and deletions, these can be collected in the following way:

Email your co-authors with the change, and ask them to reply to your email confirming that they agree to these changes. Once you have collected these replies, please combine all of the co-authors' email responses in one document and upload this file to your submission.

Your paper is now available for you to edit, you may access via the following link:

https://mts-cddis.nature.com/cgi-bin/main.plex?el=A2CA6UZA6D2dOU5F1A9ftdqTfhMrqgyuhG60SwflYg7QZ

(Press/Click on the above link to be automatically sent to the web page.)

Please make the correction(s) as specified above and resubmit your paper following the same steps as before.

If you have any questions please feel free to contact us.

Sincerely,

Editorial Office
Cell Death & Disease
cddisease@springernature.com

Our flexible approach during the COVID-19 pandemic
If you need more time at any stage of the peer-review process, please do let us know. While our systems will continue to remind you of the original timelines, we aim to be as flexible as possible during the current pandemic.

This email has been sent through the NPG Manuscript Tracking System NY-610A-NPG&MTS

Confidentiality Statement:

This e-mail is confidential and subject to copyright. Any unauthorised use or disclosure of its contents is prohibited. If you have received this email in error please notify our Manuscript Tracking System Helpdesk team at http://platformsupport.nature.com .
Details of the confidentiality and pre-publicity policy may be found here http://www.nature.com/authors/policies/confidentiality.html
Privacy Policy | Update Profile

From: Vern Dolinsky Vernon.Dolinsky@umanitoba.ca
Subject: Re: CDDIS-21-1957RRR Initial Quality Check
Date: November 4, 2021 at 8:11 PM
To: Joe Gordon Joseph.Gordon@umanitoba.ca

VD

I confirm that I approve of the inclusion of additional authors.

Vernon Dolinsky

From: Joe Gordon
Sent: Thursday, November 4, 2021 10:26 AM
To: Matthew Martens; nivedi87@gmail.com; Nguyen Lucas; Donald Chapman; Elizabeth Henson; Bo Xiang; landonfalk; arielysm@bcm.edu; Sunil Rattan; Jared Field; Philip Kawalec; Spencer Gibson; Richard Keijzer; Ayesha Saleem; Grant Hatch; Christine Doucette; Jason Karch; Vern Dolinsky; Ian Dixon; Adrian West
Subject: Fwd: CDDIS-21-1957RRR Initial Quality Check

Hello everyone,

During the review process the author list for Matt's paper changed (see below). Most notably, Jared Field and Philip Kawalec were added for their assistance with the flow cytometry experiments.

The paper has been accepted, but the journal (Cell Death and Disease) needs confirmation that all authors agree to this authorship change.

Please reply as soon as you can with a simple "Confirm" and I will compile and upload.

Thank you for all your support.

Joe.

Matthew D. Martens[1,8], Nivedita Seshadri[2,8], Lucas Nguyen[8], Donald Chapman[8], Elizabeth S. Henson[3,10], Bo Xiang[4,8], Landon Falk[2,9], Arielys Mendoza[12], Sunil Rattan[4,11], Jared T. Field[1,8], Philip Kawalec[1,8], Spencer B. Gibson[3,10], Richard Keijzer[5,9], Ayesha Saleem[7,8], Grant M. Hatch[4,8], Christine A. Doucette[2,8], Jason M. Karch[12], Vernon W. Dolinsky[4,8], Ian M. Dixon[2,11], Adrian R. West[2,9], Christof Rampitsch[13], and Joseph W. Gordon[1,6,8,*]

> Begin forwarded message:
>
> **From:** cddisease@springernature.com
> **Subject:** **CDDIS-21-1957RRR Initial Quality Check**
> **Date:** November 4, 2021 at 9:36:18 AM ADT
> **To:** joseph.gordon@umanitoba.ca
> **Reply-To:** cddisease@springernature.com
>
> **Caution:** This message was sent from outside the University of Manitoba.
>
> Dear Dr Gordon,
>
> In checking in your manuscript submitted to Cell Death & Disease it has come to our attention that the following must be addressed before we can proceed.
>
> 1. Please make sure you include a data availability statement in your Article file under its own subheading.
>
> 2. Please make sure all tables and figures are cited in the main text, in numerical order
>
> 3. It has come to our attention that your most recent author list differs from the one in your original submission.
>
> Please request agreement from all authors including additions and deletions, these can be collected in the following way:

Email your co-authors with the change, and ask them to reply to your email confirming that they agree to these changes. Once you have collected these replies, please combine all of the co-authors' email responses in one document and upload this file to your submission.

Your paper is now available for you to edit, you may access via the following link:

https://mts-cddis.nature.com/cgi-bin/main.plex?el=A2CA6UZA6D2dOU5F1A9ftdqTfhMrqgyuhG60SwflYg7QZ

(Press/Click on the above link to be automatically sent to the web page.)

Please make the correction(s) as specified above and resubmit your paper following the same steps as before.

If you have any questions please feel free to contact us.

Sincerely,

Editorial Office
Cell Death & Disease
cddisease@springernature.com

Our flexible approach during the COVID-19 pandemic
If you need more time at any stage of the peer-review process, please do let us know. While our systems will continue to remind you of the original timelines, we aim to be as flexible as possible during the current pandemic.

This email has been sent through the NPG Manuscript Tracking System NY-610A-NPG&MTS

Confidentiality Statement:

This e-mail is confidential and subject to copyright. Any unauthorised use or disclosure of its contents is prohibited. If you have received this email in error please notify our Manuscript Tracking System Helpdesk team at http://platformsupport.nature.com . Details of the confidentiality and pre-publicity policy may be found here http://www.nature.com/authors/policies/confidentiality.html
Privacy Policy | Update Profile
